# Estimating stimulus-processing time via position slopes in visual search

**DOI:** 10.3758/s13414-026-03325-5

**Published:** 2026-10-13

**Authors:** Talke Michaelsen, Markus Janczyk, Heinrich R. Liesefeld

**Affiliations:** Department of Psychology, University of Bremen, Hochschulring 18, 28359 Bremen, Germany

**Keywords:** Object processing speed, Visual search slopes, Clump scanning, Inefficient search, Systematic search

## Abstract

**Abstract:**

Existing approaches to measure the time needed to process a single stimulus have confounded the measure with other time-consuming processes, such as initiation of the response, and neglect the massively parallel processing abilities of the visual system. Conversely, traditional set-size manipulations in visual search yield flawed estimates of stimulus-processing time, mainly because the number of actually processed stimuli is typically unknown. We have recently developed the *systematic-search paradigm*, a visual-search paradigm that overcomes this limitation and potentially provides a valid measure of stimulus-processing time. Its core idea is to bring the order of search under experimental control, so that the number of processed stimuli is determined by the position of the target on the current trial. We then extract the slope of the linear function relating target position (and therefore the number of processed stimuli) to response times. With this approach, all extraneous processing times are relegated to the intercept of the function. The present study provides a further validation of this slope-measure by showing that it reflects the complexity of the visual stimuli (Exp. 1), but is robust against changes in the complexity of the response (Exp. 2). All raw data, analysis code, preregistrations, and research materials are available online (https://osf.io/42mxh/).

**Open practices statement:**

In both experiments, we report how we determined our sample size, all data exclusions (if any), all manipulations, and all measures in the study. The experiments’ designs and analyses were preregistered. All trial-level raw data, analysis code, preregistrations and research materials are available online (https://osf.io/42mxh/). Direct links to the preregistrations are https://osf.io/tbk8s/ (Exp. 1) and https://osf.io/va98q/ (Exp. 2).

**Supplementary Information:**

The online version contains supplementary material available at https://doi.org/10.3758/s13414-026-03325-5.

Measuring how long it takes to process a stimulus is essential for many scientific and applied questions spanning many areas of psychology. For example, stimulus-processing time might serve as an index of information load which could be used to predict performance at later stages of cognition (Alvarez & Cavanagh, 2004; Lavie, 2005), and processing speed, the inverse of processing time, is believed to be a core aspect of intelligence and cognitive aging (Jensen, 1982; Owsley, 2013; Salthouse, 2005).

At first, one would think that stimulus-processing time is trivially easy to measure: Just present a stimulus and measure how long it takes a participant to react to it. Unfortunately, this approach is unsatisfying, because all kinds of processes delay these responses in addition to stimulus processing proper. The time needed to physically execute the response can be removed from this estimate by measuring the time until the (hand or eye) movement starts (Jensen, 1982; Kirchner & Thorpe, 2006). Even response preparation can be removed by a slightly more sophisticated procedure: Present a stimulus for a controlled amount of time and then replace it by an effective mask to erase the stimulus from iconic memory (e.g., Bacon-Macé et al., 2005). In this paradigm, stimulus processing time could be defined either as the shortest presentation time that yields above chance performance or as the time from which on performance does not further increase. Leaving aside difficulties of creating an effective mask for the stimulus type of interest or deciding which performance level to focus on, this approach still confounds time needed for realizing that the stimulus is shown and for initiating the processing. One reason to believe that this contributes to the estimated times is that the system's readiness for processing underlies a certain rhythm to which the stimulus presentation is not typically synchronized (Iemi & Busch, 2018; Ruzzoli et al., 2019), so that some time passes after stimulus onset before the system is ready to process the incoming information on most trials. More fundamentally though, only the earliest (perceptual) stages of processing are interrupted by the mask, whereas a full “processing” of the stimulus (e.g., until categorization) can continue based on the (partial) visual information processed before mask onset (a more nuanced treatment of what “stimulus processing” actually means is provided in the General Discussion). Finally, any estimate of processing time derived from the presentation of a single stimulus would do injustice to the visual systems’ massively parallel processing capabilities. For example, if a single stimulus is processed in 50 ms and three concurrently presented stimuli are processed in 75 ms, arguably, 25 ms is the better estimate of stimulus-processing time and the 50-ms estimate from the single-stimulus condition would overestimate processing time and, thus, underestimate the processing capabilities of the visual system. From this perspective, many previous approaches are biased to underestimate stimulus-processing time, because only one stimulus is shown at a time.

Attempts to measure stimulus-processing time with larger arrays of stimuli provide the opportunity to demonstrate the visual system’s full potential. For example, Bergen and Julesz (1983) varied the number of stimuli in a visual-search array (*set size*) and estimated processing time from the slope of the function relating this number to overall processing time on the assumptions that “the observer searches at random, but never inspects the same element twice” (p. 698). An additional common assumption is that search ends once the target is found (Treisman & Gelade, 1980). If this serial, self-terminating search model was to hold, one could estimate the time needed for processing each nontarget (*b*) from median or mean response times (mRTs) in typical visual-search tasks using the equations ${RT}_{abs}(s) ={a}_{1} + b \cdot s$ on target-absent trials and ${RT}_{pres}(s) = {a}_{2} + b \cdot s/2$, with *s *standing for set size and *a*_1_/*a*_2_ capturing all non-search-related processing times in target-absent and target-present trials, respectively. However, these assumptions are certainly wrong (Horowitz & Wolfe, 1998; Horstmann et al., 2020; Liesefeld & Müller, 2020; Wolfe, 2021) and, consequently, the estimate of processing time obtained from these set-size slopes is not valid.

There are two classes of problems with using set-size slopes as measures of stimulus-processing time. First, set-size manipulations are typically confounded with either eccentricity or stimulus density. If density is kept constant, search arrays with more stimuli need to occupy a larger amount of space and the target is on average farther away from fixation (at higher eccentricity) than with small arrays. Due to the organization of the human visual system, stimuli presented in the periphery are more difficult to process, which affects error rates and mRTs (Carrasco et al., 2004). If eccentricity is kept constant, the average distance between individual stimuli decreases with the number of stimuli and this increase in density increases the local feature contrast (salience). Target salience is a major contributor to search times according to almost all current models of visual search (Gaspelin et al., 2025; Theeuwes, 2023; Wolfe, 2021).

Second, the number of stimuli processed can be calculated only under the very simple model described above, which assumes that all stimuli in the display are individually processed on target-absent trials and half the display is processed on average on target-present trials. However, according to current models of search, the number of stimuli processed in a display depends on various cognitive mechanisms. Most notably, the core assumption of the most influential model of visual search, Guided Search, is that participants preferentially process stimuli that receive a high weight on a preattentive priority map (Wolfe, 2021). The purpose of this map precisely is to inform (guide) search and thus avoid the processing of a (typically unknown) number of nontargets. Similarly, Lleras et al. (2020) assume that only objects that are similar to the searched-for target (candidates) are attentionally processed. There is also strong evidence that participants often prematurely end search with a target-absent response (Wolfe & Van Wert, 2010), so that they do not process all stimuli in target-absent displays and sometimes miss the target on target-present displays (thus biasing correct-response target-present mRTs towards trials on which the target was found early on). Both mechanisms yield a nonquantifiable underestimation of stimulus-processing time, because less stimuli are actually searched than assumed by the oversimplified serial, self-terminating search model. A bias in the opposite direction occurs, because participants do not (perfectly) track which stimuli were already searched and therefore reinspect already rejected stimuli (Horowitz & Wolfe, 1998; Horstmann et al., 2020). Readers are invited to model these and other influences on set-size slopes for themselves by using the interactive spreadsheet available online (https://github.com/Liesefeld/searchSlopes).

Recently, Michaelsen et al. (2024) developed a new experimental paradigm, which they called *systematic search*, that holds the potential to derive a better measure of visual processing time from inefficient visual search. They presented participants with a circular arrangement of stimuli in a task that induced a search in clockwise order starting at the 1 o’clock position. By presenting the response-relevant target stimulus at different positions along the circle, they obtained estimates of how long it takes until the target is processed and responded to at each of seven possible positions (for a similar design discussed in detail in the “Discussion” section, see Horowitz et al., 2009; Wolfe et al., 2000). They then regressed measured mRTs on target position (*p*) and estimated the slope (*b*) and the intercept (*a*) of that function. That the mRT data is well described by a linear function of target position was evidenced by the high amount of explained variance in mRTs (*R*^2^ > .99 for each individual participant). The beauty for the present purposes now is that the *position slope* (*b*) provides an estimate of the time needed to process each nontarget stimulus (stimulus-processing time). This is most evident if expressed as follows^1^:

1 $$mRT\left(p\right)=a+b\cdot \left(p-1\right).$$

Times for initiating processing after display onset, processing the target, and response selection and execution are all captured by the intercept term (*a*), as would be the case for *a*_1_ and *a*_2_ in the above formulae if the simple serial, self-terminating model applied. The time it takes to process all p-1 nontarget stimuli is reflected by b·(p-1) and, thus, each individual nontarget stimulus is processed for the duration *b*. The present study is designed to validate this interpretation of the two parameters of the linear function. In particular, Experiment 1 demonstrates that the slope is sensitive to a manipulation that unequivocally increases the difficulty of processing each nontarget stimulus, and Experiment 2 demonstrates that the intercept is sensitive to an experimental manipulation that—at face value—increases the difficulty of selecting (and/or executing) a response.

## Experiment 1: Stimulus-complexity manipulation

### Methods

#### Participants

A total of 32 students of the University of Bremen took part and received course credit or a monetary compensation of €10 per hour for their participation. All participants had normal or corrected-to-normal vision and provided informed consent prior to participation, with *n* = 23 identifying as women, *n* = 9 as men, and none identifying as nonbinary. Their average age was 25.6 years (*SD* = 6.2) and *n* = 29 were right-handed. No participant was excluded due to the preregistered criterion of an accuracy below 75%. A justification of the sample size requires details on the experimental design and is therefore provided below. For both experiments, convenience sampling was used—that is, participants were recruited through a mailing list used mostly for participant recruitment at University of Bremen. We did not gather ethnicity or race information about the participants and we asked their “Geschlecht,” which is the German word covering both sex and gender.

#### Overview

Each participant performed the systematic-search task with two stimulus conditions, a *simple-stimuli condition* and a *complex-stimuli condition,* in a blocked fashion with an extended mandatory break in between and in counterbalanced order. Unless otherwise mentioned, the details of structure and procedure of the systematic-search task were identical to the systematic-search task in Michaelsen et al. (2024) and are described in detail below. In broad strokes, participants were presented with a search cue followed by a circular search display. The first stimulus in clockwise direction that matches this search cue was the to-be-found *search target*. *Stimulus-processing time* was operationalized as the *b* weights from linear regressions of mRTs on target position (Positions 1–7, corresponding to 1–7 o’clock on a clockface), calculated for each individual participant and condition. These position slopes and the respective intercepts were our main dependent variables of interest.

#### Preregistered hypotheses

We predicted position slopes significantly larger than 50 ms/stimulus in both conditions, as the minimum time to shift attention from one stimulus to the next has been estimated to be 58–92 ms (Grubert & Eimer, 2016; see also Woodman & Luck, 1999). The crucial hypothesis test was a comparison of the resulting position slopes between the simple- versus complex-stimuli conditions, with the expectation that position slopes will be larger for the more complex stimuli (see https://osf.io/tbk8s).

#### Sample size

As preregistered, we applied sequential sampling with Bayes factors (*BF*s), following the recommendations by Schönbrodt and Wagenmakers (2018). The critical test was a directed paired *t* test to compare the position slopes and a *BF*_+0_ ≥ 6 acted as stopping criterion. The targeted *BF* was clearly exceeded already with our preregistered minimum of 32 participants. For details on and interpretation of *BF*s, please see the “Analyses” section.

#### Stimuli

**Fig. 1:**
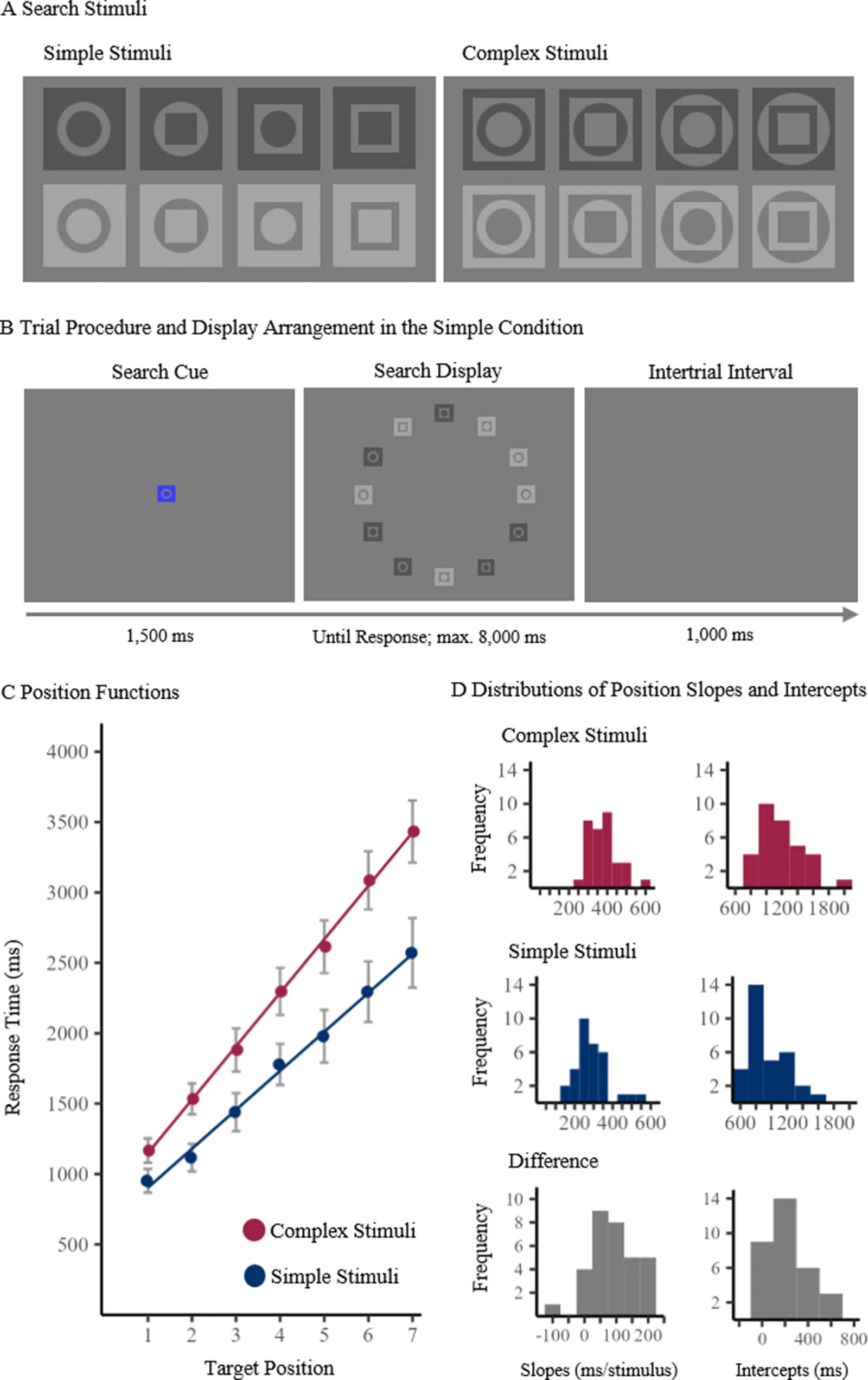
Stimuli, arrangement, and results of Experiment 1. **A** As detailed in the text, the stimuli of Experiment 1 differed in visual complexity, but not in informational content. **B** Participants first saw a search cue defining the target shape and then saw a circularly arranged search display. They had to find the first stimulus in clockwise order matching the search cue and report its luminance (dark vs. bright) via button presses. **C** Resulting mRTs averaged across participants. The main finding is that the slope for complex stimuli is steeper than that for simple stimuli. Error bars indicate between-participants 95% CIs. **D** Distribution of parameter estimates across participants. Note that the slope distributions are hardly overlapping (red vs. blue) and that almost every participant produced a shallower slope for simple stimuli (gray). Out of 32 participants only one showed a reversed pattern (~ − 100 ms) and four showed no effect (~ 0 ms). (Color figure online)

Stimuli were designed so that simple stimuli would be sufficiently complex to induce inefficient search, that is, clump scanning according to Liesefeld and Müller (2020; details on this search mode are provided in the General Discussion section “Scope of the Present Approach”), but that there was still room for complex stimuli to induce even more inefficient search. Each simple stimulus consisted of an outer gray square (1.1°) in which a square or circle with an area of 35% of the area of the outer square was embedded. Within the latter was embedded an even smaller square or circle whose area was 15% of the area of the outer square. The colors of the three shapes were such that the shapes with 100% (outer square) and 15% area appeared in the same shade of gray (light, RGB: 165, 165, 165, or dark, RGB: 85, 85, 85), while the shape with 35% area had the background shade (RGB: 125, 125, 125) and looked as if it had been cut out. This resulted in four different simple stimulus shapes (see Fig. 1A).

Each complex stimulus contained another additional circle or square whose area was 60% of the outer square. If the shape with 35% area was a circle, a square was added and vice versa. The color of the four shapes was such that the shapes with 100% and 35% area appeared in the same shade of gray (light or dark), while the shapes with 60% and 15% area had the background shade and looked as if they had been cut out. This resulted in four different complex stimulus shapes (see Fig. 1A).

In both conditions, stimuli can be discriminated by processing the two inner shapes (15% area and 35% area). The additional 60% area of the complex stimuli is not relevant for the correct identification of the stimuli. Hence, the 60% area rendered the stimuli visually more complex while maintaining a consistent informational content, without incurring limitations due to visual perception by adding finer details. Likewise, each set (simple and complex stimuli) contained an equal number of stimuli. Consequently, the simple and complex stimuli did not differ in terms of informational content.

#### Arrangement

Distance between eyes and the screen was kept constant at 60 cm via a chin-and-forehead rest. Each search array in both conditions consisted of 12 stimuli arranged analogously to the structure of a clock on an imaginary circle of diameter 9.68°, resulting in an interstimulus distance of 2.51° (center to center; see Fig. 1B). There were three types of stimuli: ten nontargets, one target, and one stimulus that we refer to as a “lure.” The lure matched the target definition except that it was always presented on a position after the target (when continuing in a clockwise direction). It is crucial for reliably inducing a predictable search order (Michaelsen et al., 2024). As we spared the 12 o’clock position and presented the lure 2–4 positions after the target, the target could appear only on Positions 1–7 and the lure could appear only on Positions 3–11. The shape defined the target (and lure), whereas the luminance defined the response. Target and lure luminance were fully independent and balanced within each block (see “Experimental Design” section below), so that lure luminance was not informative of target luminance. Within displays, nontarget luminance was randomly assigned, with the restriction that each luminance (light vs. dark, including the target and the lure) occurred exactly six times. All display configurations occurred unpredictably intermixed.

#### Procedure

Each trial started with a 1,500-ms presentation of a blue search cue, isoluminant to the background, in the center of the screen. This search cue indicated the shape of the target on the current trial. Then, the search array was shown and stayed on screen until response, with a maximum duration of 8,000 ms. Participants were informed about the fact that Position 12 (12 o’clock) never contained a target and that the lure luminance was uninformative of target luminance. Full instructions are provided in Supplement A. The intertrial interval was 1,000 ms. Participants were to respond to the luminance of the target (light vs. dark) by pressing one of two keys (D vs. L; balanced) with their index fingers as quickly as possible. In case of an incorrect response, the feedback “Leider falsch!” (German for “Unfortunately, wrong!”), and in case of a timeout, the feedback “Leider zu langsam!” (“Unfortunately, too slow!”) was displayed for 500 ms.

#### Experimental design

Each combination of target position (1–7) × target–lure distance (2–4 positions) × target luminance (light vs. dark) × lure luminance (light vs. dark) × target (and lure) shape (4 different shapes) occurred once in each stimulus-complexity (simple vs. complex) block. All other values were drawn at random without counterbalancing with the restriction that nontarget shapes were randomly drawn from the three remaining versions after excluding the respective target shape.

Each of the two main-phase blocks consisted of 336 trials. After each 112 trials, there was an extended break and feedback on accuracy was provided. Within each of these 112-trial epochs, there were two self-paced shorter breaks without feedback after 37 and 74 trials. Before each stimulus-complexity block, participants went through a familiarization phase to ensure they understood the distinction between target and lure stimuli. They were shown example displays and had to point-and-click on the target in each display. An experimenter was available to assist if necessary. To confirm understanding, participants stayed in this phase until they correctly selected the target in at least five out of six trials. Afterward, participants repeatedly performed a training block (20 trials) until they committed less than 20% errors. The training was performed an average of 1.2 times (*SD* = 0.45, *min* = 1, *max* = 3) for simple stimuli and 1.3 times (*SD* = 0.8, *min* = 1, *max* = 5) for complex stimuli. That is, most participants passed the 20% criterion already on the first attempt. After each stimulus-complexity block, the experimenter conducted a standardized interview on the participants’ search strategy.

#### Analyses

Data were analyzed using R (Version 4.3.1; R Core Team, 2023) and the packages *readr* (Version 2.1.5; Wickham et al., 2024), *tidyverse* (Version 2.0.0; Wickham et al., 2019), *schoRsch* (Version 1.10; Pfister & Janczyk, 2016), *car* (Version 3.1.2; Fox & Weisberg, 2011), *lsr* (Version 0.5.2; Navarro, 2015), *BayesFactor* (Version 0.9.12.4.5; Morey et al., 2023), *ggplot2* (Version 3.4.4; Wickham, 2016), and *jtools* (Version 2.2.2; Long, 2022). The analyses below focus on mRTs. In particular, we calculated the median correct response times per participant and condition (*design cell*) and entered these into inferential statistical analyses or ran linear regressions on individual mRTs with position as a linear predictor (see Supplement B). Intercepts and *b* weights (i.e., slopes) from these regressions were also entered into statistical tests. For the analyses on position slopes against fixed values and the comparison of slopes, we report directed tests (one-tailed, as indicated), because we expected steep positive slopes > 50ms/stimulus in both conditions and larger slopes in the complex-stimuli condition. Analyses on error rates are reported in Supplement C.

Bayes Factors (*BF*s) were computed using the standard JZS Cauchy prior with an *r*_scale_ of 0.707. We report *BF*_+0_ for directed and *BF*_10_ for nondirected tests. We employed Jeffreys’ criterion (van Doorn et al., 2021) for categorizing evidence strength. *BF*s in the range of 1 to 3 indicate weak, 3 to 10 suggest moderate, and values greater than 10 indicate strong evidence for the alternative hypothesis. Conversely, *BF*s between 0.33 and 1 signify weak, 0.10 to 0.33 indicate moderate, and values below 0.1 suggest strong evidence for the null hypothesis.

#### Ethics

All procedures performed in this study are in accordance with the institutional and national ethical standards for research involving human participants and are thereby exempt from dedicated ethics approval, because they involve no deception and no physical or mental risks.

### Results

For simple stimuli, there was no significant effect of stimulus-condition order on mRT position slopes, *t*(30) = 1.99, *p* = .056, *d* = 0.70, *BF*_10_ = 1.46, or intercepts, *t*(26.14) = 1.47, *p* = .153, *d* = 0.52, *BF*_10_ = 0.76, nor for complex stimuli, *t*(20.96) = 1.23, *p* = .233, *d* = 0.43, *BF*_10_ = 0.60, *t*(30) = 1.08, *p* = .289, *d* = 0.38, *BF*_10_ = 0.52. Accordingly, and because we were not interested in the effect of condition order, we combined the two groups.

For both simple and complex stimuli mRTs were well described by a linear function of position. For simple stimuli, individual position slopes varied across participants from *b* = 154 ms/stimulus to *b* = 547 ms/stimulus with a mean position slope of $\overline{b }$ = 289 ms/stimulus (*SD* = 86). This average position slope was significantly larger than our preregistered criterion of 50 ms/stimulus, *t*(31) = 15.74, *p* < .001, one-tailed against 50, *d* = 2.78, *BF*_+0_ = 4.56 × 10^13^. For complex stimuli, individual position slopes varied across participants from *b* = 261 ms/stimulus to *b* = 603 ms/stimulus with a mean position slope of $\overline{b }$ = 383 ms/stimulus (*SD* = 76). This average position slope was again significantly larger than our preregistered criterion, *t*(31) = 24.87, *p* < .001, one-tailed against 50, *d* = 4.40, *BF*_+0_ = 1.44 × 10^19^. All individual model fits as quantified by *R*^2^ were almost perfect for simple, $M_{R^2}=.98$, $SD_{R^2}=.02$, and complex stimuli, $M_{R^2}=.98$, $SD_{R^2}=.01$. As predicted, slopes for complex stimuli were significantly larger than those for simple stimuli on average, *t*(31) = 7.54, *p* < .001, *d* = 1.33, *BF*_+0_ = 1,856, as well as for almost each individual participant (Fig. 1D, lowest row). Position functions for individual participants are displayed in Supplement B.

Intercepts for simple stimuli varied across participants from *a* = 549 ms to *a* = 1,557 ms with a mean intercept of $\overline{a }$ = 957 ms (*SD* = 225). Intercepts for complex stimuli varied across participants from *a* = 779 ms to *a* = 1,911 ms with a mean intercept of $\overline{a }$ = 1,181 ms (*SD* = 265). Intercepts for complex stimuli were significantly larger than intercepts for simple stimuli, *t*(31) = 7.13, *p* < .001, *d* = 1.26, *BF*_10_ = 50.2. In sum, strong linear position effects confirm that we successfully induced the ordered search mode as in Michaelsen et al. (2024) and the effect of stimulus complexity confirms our main hypothesis that these slopes reflect stimulus-processing time (see Fig. 1C and D). Furthermore, an exploratory (yet preregistered) analysis revealed an additional effect of stimulus complexity on the intercept, which likely is related to target processing (see "General Discussion"). The structured interviews indicated that a serial processing of one stimulus after the other was the predominant subjectively felt search strategy.

## Experiment 2: Response-complexity manipulation

A core advantage of the here-used linear-regression approach over other approaches for measuring stimulus-processing time is that all times unrelated to processing of the nontargets are relegated to the intercept, so that the slope provides a pure measure of stimulus-processing time. To demonstrate that this purification is indeed achieved, we manipulated the complexity of the response in Experiment 2. In particular, we compared a two-alternative response to a four-alternative response, with the expectation that this manipulation would affect only the intercept of the linear function relating target position and mRTs.

### Methods

#### Participants

From a total of 32 participants, none was excluded due to the preregistered criterion of an accuracy below 75%. Fourteen of the participants had already participated in Experiment 1. All participants had normal or corrected-to-normal vision and provided informed consent prior to participation. They were 26.2 years (*SD* = 6.1) old on average, with *n* = 24 identifying as women, *n* = 8 as men, and none identifying as nonbinary, with *n* = 27 being right-handed.

#### Preregistered hypotheses

Again, we predicted position slopes significantly larger than 50 ms/stimulus in both conditions. Moreover, the crucial hypothesis test was a comparison of the resulting intercepts between the two response-type conditions (simple vs. complex), with the expectation that intercepts will be larger for the more complex response (see https://osf.io/va98q).

#### Sample size

The critical test was a directed paired *t* test to compare the intercepts and a *BF*_+0_ ≥ 6 acted as stopping criterion. The targeted *BF* was clearly exceeded already with our preregistered minimum of 32 participants.

#### Stimuli

**Fig. 2:**
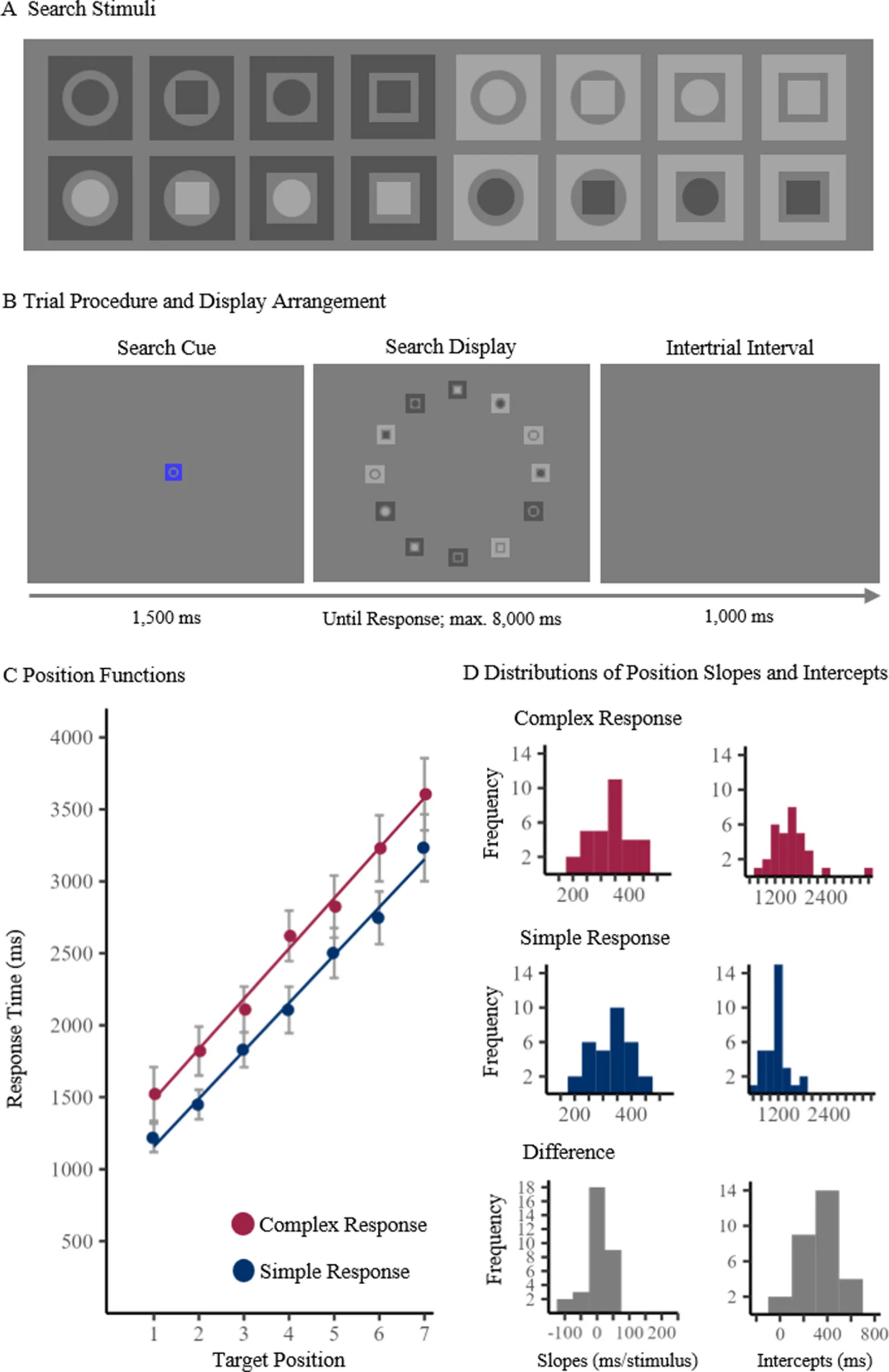
Stimuli, arrangement, and results of Experiment 2. **A** The same set of stimuli was used in both conditions of Experiment 2. The stimuli in the upper row are the simple stimuli of Experiment 1 and the stimuli in the lower row were constructed by inverting innermost luminance (from dark to bright or vice versa). **B** The simple-response task was identical to Experiment 1, except that after finding the target participants report the luminance of the outer square (two response options). In the complex-response condition, the luminance of the outer square defined the hand and the luminance of the inner shape defined the finger to respond with (four response options). **C** Resulting mRTs averaged across participants. The main finding is that there is an effect on the intercept although the slope for both tasks is comparable (the lines are almost parallel). Error bars indicate between-participants 95% CIs. **D** Distribution of parameter estimates across participants. Note that the slope distributions are almost identical (red vs. blue), whereas the intercept distributions differ between the simple-response and complex-response conditions. (Color figure online)

Stimuli for both response-complexity conditions resembled the simple stimuli from Experiment 1, with the exception that the color of the three shapes was such that the shapes with 100% (outer square) and 15% area appeared in dark (RGB: 85, 85, 85) or light (RGB: 165, 165, 165) gray color, whereby all combinations (dark/dark, dark/light, light/dark, light/light) occurred with equal probability. This resulted in four different stimulus shapes, each of them with four different color combinations (see Fig. 2A).

#### Arrangement

Lure and nontarget luminance combinations were randomly assigned as described for Experiment 1, with the additional restriction that, for both the outer (100% area) and inner (15% area) shapes (including the target), each luminance (light and dark) occurred exactly six times in each area in each search display.

#### Experimental design and procedure

Design and procedure were identical to those of Experiment 1, except that instead of stimulus complexity, the two blocks differed in response complexity. In the 336 simple-response trials, participants were to respond to the luminance of the outer square (100% area) of the target by pressing as quickly as possible one of two keys (D vs. L; balanced) with their index fingers.

In the 336 complex-response trials, the participants used the keys S and D with middle and index finger of their left hand and the keys K and L with index and middle finger of their right hand. To decide which key to press, they needed to take two decisions. The luminance of the outer square (100% area) defined the hand (left vs. right) and the luminance of the inner shape (15% area) defined the finger (index vs. middle finger) to respond with. The assignment of the respective hand and finger to the luminance combination was fully counterbalanced across four groups. Participants performed a familiarization phase as described for Experiment 1. The training was performed an average of 1.3 times (*SD* = 0.58, *min* = 1, *max* = 3) for simple responses and 2.1 times (*SD* = 1.4, *min* = 1, *max* = 7) for complex responses. That is, in the simple-response condition, most participants passed the 20% criterion already on the first attempt, while in the complex-response condition, the majority of the participants needed two or more attempts.

#### Analyses

For the mRT analyses on position slopes against fixed values and the comparison of intercepts, we report directed tests (one-tailed, as indicated), because we expected steep positive slopes > 50ms/stimulus in both conditions and larger intercepts in the complex-response condition.

### Results

There was no significant effect of condition order on mRT position slopes, *t*(30) = 1.12, *p* = .272, *d* = 0.40, *BF*_10_ = 0.54, or intercepts, *t*(30) = 1.66, *p* = .107, *d* = 0.59, *BF*_10_ = 0.95, for simple responses nor complex responses, *t*(30) = 1.68, *p* = .104, *d* = 0.59, *BF*_10_ = 0.97, *t*(30) = 0.53, *p* = .597, *d* = 0.19, *BF*_10_ = 0.38. Accordingly, and because we were not interested in the effect of condition order, we combined the two groups.

For both the simple and the complex responses, mRTs were well described by a linear function of position (Fig. 2C and Supplement B). For simple responses individual position slopes varied across participants from *b* = 205 ms/stimulus to *b* = 527 ms/stimulus with a mean position slope of $\overline{b }$ = 336 ms/stimulus (*SD* = 74). The average position slope was significantly larger than our preregistered criterion of 50 ms/stimulus, *t*(31) = 21.73, *p* < .001, one-tailed against 50, *d* = 3.84, *BF*_+0_ = 3.14 × 10^17^. For complex responses, individual position slopes varied across participants from *b* = 196 ms/stimulus to *b* = 545 ms/stimulus with a mean position slope of $\overline{b }$ = 339 ms/stimulus (*SD* = 76). This average position slope was again significantly larger than our preregistered criterion, *t*(31) = 21.41, *p* < .001, one-tailed against 50, *d* = 3.78, *BF*_+0_ = 2.08 × 10^17^. All individual model fits as quantified by *R*^2^ were almost perfect for simple responses, $M_{R^2}=.97$, $SD_{R^2}=.03$, and for complex responses, $M_{R^2}=.96$, $SD_{R^2}=.07$. Moreover, position slopes did not significantly differ between simple and complex responses, *t*(31) = 0.48, *p* = .637, *d* = 0.08, *BF*_10_ = 0.26. These results indicate that participants searched in the desired clockwise order and that our manipulation of response complexity did not affect search speed.

Intercepts for simple responses varied across participants from *a* = 664 ms to *a* = 1,750 ms with a mean intercept of $\overline{a }$ = 1,156 ms (*SD* = 246). Intercepts for complex responses varied across participants from *a* = 869 ms to *a* = 3,337 ms with a mean intercept of $\overline{a }$ = 1,581 ms (*SD* = 462). As predicted, intercepts for complex responses were significantly larger than intercepts for simple responses, *t*(31) = 6.58, *p* < .001, *d* = 1.16, *BF*_+0_ = 1,697. In sum, the response-complexity manipulation led to the expected change in intercept, while search speed remained almost unaffected (see Fig. 2C–D). The structured interviews indicated that a serial processing was the predominant subjectively felt search strategy. Interestingly, more participants (*n* = 7) reported having processed more than one stimulus at a time in the simple-response condition than in the complex-response condition (*n* = 2). If participants had indeed processed more stimuli at the same time (without slowing down the processing of individual stimuli), the increased processing efficiency should have resulted in lower search slopes for simple compared with complex responses, which was not confirmed by the objective performance data.

## General discussion

We have proposed and validated a new approach to measure stimulus-processing time via performance on a modified visual-search task. The approach is to induce a consistent search order via our novel systematic-search paradigm (Michaelsen et al., 2024) and then determine the slope of the linear function relating the search target’s position in the search array to mRTs (position slope). On trials with the target on position *n*, *n* – 1 nontarget stimuli are processed before the target is reached, so that this position slope provides an estimate of the time needed to process each nontarget stimulus (i.e., stimulus-processing time). If the relationship between mRT and target position (i.e., number of processed stimuli before the target is reached) is well described by a linear function and if the slope of this function is a suitable measure of stimulus-processing time, it should be affected by an increase in stimulus-processing difficulty, but not by response-related processes. Indeed, this is what we demonstrate here: A manipulation of the complexity of the visual stimuli affected the slope (Exp. 1), but a manipulation of the complexity of the response did not (Exp. 2). This corroborates our claim that this position slope is a suitable index of stimulus-processing time.

### Influences on the intercept: Times unrelated to nontarget processing

All time-consuming processes unrelated to the processing of nontargets affect the intercept of the analyzed linear function. Ordered by timing, these include at least five steps (a) registering the onset of the search array, (b) starting the search process, (c) processing the target shape, (d) processing and deciding on the target luminance, and (e) programming/execution of the manual response. The intercept would also bind (f) technical delays related to the screen’s refresh rate and the polling of the response device.

We found an effect of the respective complexity manipulation on the intercept in both experiments. While the effect on the intercept of Experiment 2 was an intended and selective consequence of our manipulation of response complexity, the effect of stimulus complexity in Experiment 1 needs further explanation. Obviously, by manipulating stimulus complexity, not only the processing of the nontargets was affected, but the processing of the target as well. Furthermore, the time to process the target is unlikely to be the same as the time to process the nontargets for various reasons, so that we cannot simply subtract nontarget-processing time to remove the influence of target processing from the intercept. For example, given that it is much more likely to encounter a nontarget, each decision on whether the currently presented stimulus is a target or a nontarget might be biased for the latter, thus resulting in faster “nontarget” decisions than “target” decisions. On this background, we tentatively interpret the effect of stimulus complexity on the intercept in Experiment 1 as an effect on the difficulty of target processing (point c from the above list).

From a purely mathematical perspective, one might expect that the intercept always changes along with the slope, so that this effect is somewhat trivial and does not require further explication. For example, when mRT averaged across positions is constant and the slope increases, the intercept must decrease. This is not the reason for the observed effect on the intercept in Experiment 1, however. First, average mRT was not constant and instead of decreasing, the intercept increased. Second—and more importantly—from the perspective of the underlying cognitive model, the intercept is the time point at which zero nontargets are processed and, thus, nontarget processing time cannot affect the intercept.

### Influences on the slope: How to define “stimulus processing”?

Our index of stimulus-processing time (the position slope) includes the time to disengage, shift, and reengage attention (Fukuda & Vogel, 2011; Horstmann et al., 2020; Sauter et al., 2021; Toffanin et al., 2011; Yamaguchi et al., 2018; Zivony & Lamy, 2018). Shifting of attention is unlikely to be affected by the specific stimuli used, thus simply adding a constant to the estimate of stimulus-processing time (if it is time-consuming at all). However, it appears plausible that dis-engagement and re-engagement of attention depend on stimulus complexity as well as processing proper, thus contributing variance to our measure of stimulus-processing time. In this regard, it is a theoretical possibility that two stimulus types are processed equally fast, but one of them produces longer dis-engagement or re-engagement times. Depending on the definition of stimulus-processing time, it might actually be desirable to include these times into the estimate of stimulus-processing time, because disengagement and reengagement can be viewed as central components of stimulus processing.

Relatedly, the time it takes to decide that a nontarget is not the target (“processing proper”) would likely depend on its similarity to the target (its “discriminability”; cf. Duncan & Humphreys, 1989). From an evidence-accumulation perspective (e.g., Ratcliff, 1978), target similarity of the currently inspected nontarget might affect the accumulation rate and the (preaccumulation) expected similarity might affect the decision threshold. The more fundamental insight is that there might not be “stimulus processing” as such that would depend exclusively on the processed stimulus. Rather, stimulus processing always has a purpose. In the present paradigm, this purpose is the comparison with a target or nontarget template and stimulus-processing time therefore must depend on the (expected) nontarget-target-similarity.^2^

Finally, our paradigm would allow for parallel processing of multiple stimuli if this is something the visual system is able to do (see, e.g., the car-wash idea in Wolfe, 2007). Does that mean that we underestimate stimulus-processing time, because processing a single stimulus would take longer than indicated by our position-slope estimate? Again, the answer depends on how we define stimulus-processing time. As argued in the introduction, we think that processing three stimuli in 75 ms is faster than processing one stimulus in 50 ms and therefore paradigms that do not allow for parallel processing by design would overestimate stimulus-processing time from this perspective. We argue that all three points (dis-engagement/re-engagement, processing purpose, and parallel processing) and the potential involvement of eye movements (discussed in the next section) do not invalidate our measure of stimulus-processing time, but must be considered as aspects of stimulus processing when interpreting our measure.

### Potential contribution of eye movements

As distinguishing our targets and nontargets requires a relatively high visual resolution, eye movements might be needed despite the relatively low eccentricity (4.84°). If each stimulus needs to be fixated, the time it takes to make a saccade would set a lower limit on the position slope. This possibility has already been discussed and rejected by Michaelsen et al. (2024) based on the observations that neither increasing stimulus eccentricity (Michaelsen et al., 2024) nor enforcing fixation (Horowitz et al., 2009) had noticeable effects on position slopes. However, it is still interesting to compare position slopes to estimates of minimal saccade rates around 250 ms/stimulus (Schütz & Steward, 2025; Wolfe, 2020) or 200 ms/stimulus (Fischer & Weber, 1993). The position slopes averaged across participants that we have observed so far range between 164 ms/position and 383 ms/position and slopes by individual participants range between 85 and 603 ms/position. The upper range of slopes comes from the complex stimuli of the present Experiment 1, which are specifically designed to induce slow search, whereas the lower range comes from an experiment using stimuli more commonly employed in the current visual-search literature (Liesefeld et al., 2025). Thus, the smaller slopes are too shallow to be produced by eye movements and the steeper slopes are obviously not clipped at the minimal rate of eye movements. Far from being fully constrained by eye movements, there obviously is stimulus-related and participant-related variance in our measure of stimulus-processing time that has the potential to inform psychological theories.

From a theoretical perspective, any time needed for dis-engaging, shifting, and re-engaging attention is reasonably subsumed under the theoretical construct of stimulus-processing time as discussed above. If that is true for covert attention, one would reasonably also include times contributed by overt attention: stimulus-processing time might as well subsume the time to physically (dis-)engage and move the eyes in situations where eye movements are a necessary component of stimulus processing, simply because eye movements are an integral part of processing these stimuli. That is, covert and overt shifts of visuo-spatial attention are not so fundamentally different as one might initially think and both are likely affected by the difficulty of processing the employed stimuli. From this perspective, eye movements are constrained by stimulus-processing time rather than our measure of stimulus-processing time being contaminated by eye movements.

### Scope of the present approach

A prerequisite for using our systematic-search paradigm for the purpose of measuring stimulus-processing time is that the search stimuli induce clump scanning as proposed by Liesefeld and Müller (2020). In this search mode, participants process one adjacent set of stimuli (i.e., a clump) after the other in a systematic order. Liesefeld and Müller assume that clump scanning is induced if the target is (expected to be) of low salience or search displays are small (only a few items). Under these conditions, clump scanning is more efficient than the alternative search mode “priority guidance.” The latter allows to constrain processing to a small set of salient stimuli that stand out on a pre-attentive priority map. This selection is an advantage only if there are many stimuli to select from and if the selection works well. Preattentive selection works well only if the searched stimulus (the target) is salient in one of a limited number of preattentive features dimensions that could potentially guide search (Wolfe & Horowitz, 2017). This implies a limitation in the scope of the present approach: it can only be used to measure stimulus-processing times in search displays with relatively low target salience.

Salience determines search efficiency during priority guidance, but target–distractor discriminability determines search efficiency during clump scanning (Liesefeld & Müller, 2023). The two are not the same constructs, but often confounded (see Zhaoping, 2008, for an impressive dissociation). For displays with high salience, target–distractor discriminability typically is high as well and target–distractor similarity is low. However, for features that are not represented preattentively, discriminability might be high (and similarity low) without the target being salient.

A complementary approach focusing on search displays with relatively high target salience or low target–distractor similarity was proposed by Buetti and Lleras (Buetti et al., 2016; Lleras et al., 2020). Their model predicts that search times in such displays increase with each additional stimulus in a logarithmic fashion and logarithmic functions fit to empirical data from homogenous displays can be used to predict empirical data from heterogenous displays, thus demonstrating the reliability of their parameter estimates and the validity of their model. Based on theoretical observations by Townsend and Ashby (1983), they interpret the logarithmic functions to reflect a fully parallel processing of all stimuli in the display. Rejecting all nontargets can result in target-absent or target-present decisions with only this first-stage parallel processing (see also Moran et al., 2016; Narbutas et al., 2017). Only if multiple potential target stimuli (candidates) remain after the rejection stage, a second stage consisting of sequential allocations of focused attention is entered. Notably, Buetti and Lleras interpret a scale parameter in their logarithmic functions as the speed at which nontargets are rejected at the parallel stage. In analogy to slopes of linear set-size functions, they refer to this parameter as the “logarithmic slope.” From the perspective of Guided Search, one could interpret the logarithmic slope as the time needed to extract sufficient information from each stimulus to build up the preattentive priority map (Wolfe, 2021). This interpretation might not suffer from the confounds discussed above: priority-map built-up might not be affected by eccentricity and density (but see Supplement B of Buetti et al., 2016). More importantly, for both parallel rejection and priority-map built-up, it is reasonable to assume that all stimuli are processed on every trial (exhaustive termination rule; see Buetti et al., 2016). This would mean that the number of processed stimuli is actually known and equivalent to the set size. Thus, if Buetti and Lleras’ theory holds, their approach would allow to measure a type of processing time in displays with low target–nontarget similarity that only require the first, parallel stage. However, this processing time would not reflect stimulus-processing time as understood here, but the time needed for rejecting a nontarget or the time needed for preattentive stimulus processing (for the purpose of building up the priority map).

If we consider Buetti and Lleras’ theory as one potential specification of priority guidance (Liesefeld & Müller, 2020), participants would rely on this search mode only if a rather low number of candidates remains after nontarget rejection, because, according to our theory, if too many candidates remain, it is more efficient to skip the time-consuming parallel step and perform (unguided) clump scanning instead (see above). From the perspective of Target-Contrast-Signal Theory (Lleras et al., 2020), one might interpret the present search displays as consisting only of candidate stimuli and would then interpret our measure of stimulus-processing time as the time of (overt and/or covert) attentive processing at the second stage. Thus, the two theories are not in competition, but focus on different modes or different stages of visual search.

### Does the systematic-search paradigm slow down processing?

Horowitz et al., (2009; see also Wolfe et al., 2000) refer to the type of search induced here as “commanded” and argue that it requires voluntary deployments of attention that are slower than deployments of attention in the default “anarchic” search. Therefore, one might argue that our search paradigm slows stimulus processing down, thus yielding an overestimation of stimulus-processing time. Indeed, using comparable search stimuli, Horowitz et al. (2009) provide estimates of 58.5 ms/stimulus with “anarchic” search and 195.9 ms/stimulus for “commanded” search in their Experiment 4.

Unfortunately, the two estimates are not comparable. Whereas the latter estimate is based on a position effect (as suggested here), the former estimate is based on a set-size effect. As detailed in the introduction, we have argued that measuring stimulus-processing time via the set-size effect in any visual-search task is not valid, mainly because even though the number of presented stimuli is known, the number of actually processed stimuli remains unknown (Liesefeld & Müller, 2020). The main advance of our new paradigm beyond this traditional approach is that it provides experimental control over the number of (nontarget) stimuli processed on each trial. In fact, getting the number of processed stimuli under experimental control is precisely why we have developed our novel systematic-search paradigm in the first place (Michaelsen et al., 2024).

Therefore, we argue that Horowitz et al.’s (2009) estimate of 58.5 ms/stimulus is an underestimation of stimulus-processing time rather than their 195.9 ms/stimulus or the estimates provided in the present paper being overestimations. There are many influences on set-size slopes (see Liesefeld & Müller, 2020) and the search displays in Horowitz et al.’s (2009) Experiment 4 differed between the two conditions, but if we pretend that the difference in estimates is only due to the number of processed stimuli, the discrepancy observed would indicate that only around 30% of the stimuli are processed in their anarchy condition. Stimuli are skipped, because only a promising subset of stimuli is processed (guidance) or because search is ended and the response is guessed before the target is found (premature quitting). The low error rate (3.7%) indicates that not much guessing took place.

From the perspective of Liesefeld and Müller’s (2020) dichotomy, assuming guidance in the anarchy condition of Horowitz et al.’s (2009) Experiment 4 means that their participants preferentially employed the search mode we call “priority guidance.” Conversely, the (somewhat) linear position slope in their command condition indicates a strong preference for the other search mode: “clump scanning.” Thus, we agree with Horowitz et al. that the two conditions likely differ in the type of search induced, but we disagree on why they differ. In the anarchy condition, targets were defined as the only mirror-reversed *S* or *P* in the display with otherwise regular *S*s and *P*s. As orientation is known to guide attention allocations (Wolfe & Horowitz, 2017), it is reasonable to assume that these mirror-reversed letters were salient enough to induce priority guidance (Liesefeld & Müller, 2021) and constrain attentive processing to only 30% of the stimuli on average. By contrast, targets in the command condition were defined as the first mirror-reversed letter in the display and all letters after the targets had a 50% chance of being mirror reversed. These later mirror-reversed letters are what we refer to as “lures.” That is, targets were no singletons and therefore less salient on their own (lower local feature contrast), but also in comparison to other stimuli in the display (*relative salience*; Constant & Liesefeld, 2021). Furthermore, the salience signals (in the orientation dimension) produced by mirror reversedness would often misguide attention (Liesefeld & Müller, 2019). Thus, in contrast to their anarchy condition, priority guidance would be highly ineffective in Horowitz et al.’s command condition, so that participants likely resorted to clump scanning (Liesefeld & Müller, 2021).

The same argument might be applied to our systematic-search paradigm, because the target is not a singleton either. However, there was only a single lure per trial, thus biasing the search mode much less than Horowitz et al.’s (2009) command condition. Still, our paradigm might slightly bias participants towards using clump scanning, because priority guidance would often result in an attention allocation to the lure and therefore reduce the efficiency of this mode. Horowitz et al. seem to assume that their command condition forces participants to use search in the intended order, but whether it does even with highly salient targets (by virtue of the many lures, we would assume) is an outstanding empirical question.

That our systematic-search paradigm (with a single lure) does not force participants to search in the intended order has been demonstrated by Michaelsen et al. (2024). They show that when the target is salient enough, priority guidance is the most efficient search mode in our systematic-search paradigm. With a red target and lure standing out among green nontargets (efficient-search condition), position functions were far from the linearly increasing functions observed in the present study, indicating that these displays were not searched in the intended order despite comparable instructions and task requirements. Nevertheless, the slight bias means that the systematic-search paradigm is non-ideal to determine which search mode is induced by the search stimuli alone and other techniques are preferable for this purpose (e.g., Davis, 2024; Lee et al., 2021). Fortunately, this does not matter much for our present goal to gain an estimate of stimulus-processing time nor does it invalidate Michaelsen et al.’s (2024) goal to gain a better estimate of search slopes. It just limits the scope of the technique to search displays that induce clump scanning in this paradigm (as can be evaluated via the position functions).

Interestingly, we believe, Horowitz et al. (2009) and our theoretical position are not as far apart as the preceding discussion might suggest. Both theoretical positions assume two search modes, only that we characterize what Horowitz et al. consider “commanded search” as “clump scanning” and what they consider the default “anarchic search” as “priority guidance.” Crucially though, we seem to disagree on whether the former mode is induced in standard search paradigms. Whereas Horowitz et al. seem to believe that it is induced by the requirement to find the first target-matching stimulus in a prespecified order (as in their command condition and our systematic-search paradigm), we argue that the choice of search mode mainly depends on the search displays (as confirmed by the results from Michaelsen et al., 2024, discussed above). In particular, if the target is of low (relative) salience and/or the number of nontargets is low, we assume that clump scanning is more efficient than priority guidance and therefore often used also in standard visual-search paradigms (see Liesefeld & Müller, 2020, for a review of empirical evidence). We assume that people usually try both modes and preferentially use the one that turns out to be most efficient in a given search situation. For example, if attention is frequently misguided to multiple lures whenever participants use priority guidance in Horowitz et al.’s (2009) command condition, they will be incentivized to use clump scanning instead. Attention is also more likely to be misguided if the target in standard visual-search paradigms is not or only weakly salient or if many nontargets are salient due to high nontarget heterogeneity. Consequently, we argue that clump scanning contributes to many standard search tasks, in particular those that induce “inefficient search” (Liesefeld & Müller, 2020; Michaelsen et al., 2024). The reason why these standard tasks produce no or only shallow linear position slopes is not that the type of search is different. Rather, the order of search is unknown and variable across participants (and maybe trials), so that the spatial position of the target does not correspond to the temporal position at which it is processed.

### Problematic aspects of nonsearch measures of stimulus-processing time

Nonsearch approaches to measuring stimulus processing time can be collectively described as measures of the time between stimulus onset and a certain reaction (a button press or the onset of a pointing response or a saccade towards the target; e.g., Kirchner & Thorpe, 2006). These approaches share the problem that they confound times for initiating task execution (e.g., due to oscillatory changes in readiness for processing; see Introduction) and times for initiating the actual response (e.g., saccade programming) with stimulus processing time. In our approach, these times are absorbed (together with potential technical delays) by the intercept. By presenting a single stimulus, they do also prevent the massively parallel processing capabilities of the visual system from improving processing time.

Furthermore, it appears likely that with such approaches, stimulus-processing time is further underestimated, because processing has often not yet finished when a response is selected. Rather, fast responses might reflect an educated guess on the stimulus identity. In Kirchner and Thorpe (2006), for example, approximately 10% of the saccades went wrong with only two directions to choose from and the faster participants made their saccades, the more errors they made. Relatedly, the latency of stimulus effects on neural responses (ERP components, single-cell activity, above-chance decoding performance; e.g., Thorpe et al., 1996) likely reflect the starting rather than the finishing times of stimulus processing and it is unclear how activity in specific brain regions relates to what researchers would consider “having processed a stimulus.” There is certainly a difference between sufficient information for an educated guess and a certain brain region reacting to features of the stimulus on the one hand and the full-blown conscious perception of that stimulus on the other hand.

### Distinction to other constructs

*Minimal exposure time* is the minimal presentation time needed to process a stimulus and estimates are sometimes as low as 13 ms (Potter et al., 2014). Minimal exposure time is not equivalent to measures of processing time, because additional processing at perceptual and nonperceptual levels might be necessary for what we and others would consider stimulus processing. Even if all necessary information can enter the visual system in 13 ms, full processing of this information by higher brain regions still requires additional time.

*Attentional dwell time* (Duncan et al., 1994) is how long a stimulus occupied attentional processing resources. Attentional dwelling has been estimated to take several hundred ms and at least up to 450 ms. Our estimates of stimulus-processing time were in the range of 150–600 ms across participants, with averages in the range 290–380 ms across experiments/conditions (see also Michaelsen et al., 2024). It appears plausible that attentional dwell-time (just as stimulus-processing time) also depends on the stimuli and task, so that these values are not directly comparable. However, Duncan et al. (1994) employed letters, which should be relatively easier (rather than harder) to process than the stimuli used here and in Michaelsen et al., (2024; see Hulleman et al., 2020). In fact, we selected stimuli specifically to induce relatively long processing times, because we considered this advantageous for the development of our task (Michaelsen et al., 2024). Importantly, attentional dwell time is not about how many stimuli can be processed in a certain amount of time (processing time), but how long each stimulus is processed (potentially in parallel with other stimuli), which is why attentional dwell times should be longer than estimates of stimulus-processing time for a given type of stimuli if there is any degree of parallel processing (see also Theeuwes et al., 2004). Thus, interestingly, stimulus-processing time and attentional dwell time might characterize related but distinguishable aspects of attentional processing.

## Supplementary Information

Below is the link to the electronic supplementary material referred to as Supplement A, B and C in the main text.

Supplementary file1 (PDF 0.99 MB)

## Data Availability

Available online (https://osf.io/42mxh/).

## Footnotes

1 Michaelsen et al. (2024) used the equation $mRT(p)={a}^{*}+b\cdot p$ [superscript added for disambiguation], which does not alter the interpretation of *b*. However, it implies that ${a}^{*}$ does encompass aspects of target processing, not the full target processing time (in contrast to *a* in the equation used here) but rather the difference between nontarget and target processing time, in addition to all other non-search time-consuming processes. Unlike in Michaelsen et al. (2024), the value of intercepts is of interest here. To improve interpretability, Eq. 1 adjusts the intercept to include the full target-processing time. The intercepts reported here can be compared with those in Michaelsen et al. (2024) by calculating ${a}^{*}=a-b$. As a side note, this makes the intercepts reported here also better comparable to Lleras et al. (2020), where mRT is assumed to be a logarithmic function of set size (s) of the form $mRT(s)=a+b\cdot \mathrm{l}\mathrm{n}(\mathrm{s})$, because for set size 1 (only the target is presented), $mRT\left(1\right)=a+b\cdot \mathrm{ln}\left(1\right)=a+b\cdot 0=a$.

2 Note the similarity to the concept of “apperception” as introduced by 18th-century philosopher Gottfried Wilhelm Leibniz, which was also central to early psychologists such as Wilhelm Wundt.

## Code availability

Available online (https://osf.io/42mxh/).
