# Supplementary material for: Estimating stimulus-processing time via position slopes in visual search
Source: Atten Percept Psychophys. 2026 Oct 13;88(7):206. doi: 10.3758/s13414-026-03325-5 (PMC13661710; doi:10.3758/s13414-026-03325-5)
Supplement: Supplementary file 1 [file 13414_2026_3325_MOESM1_ESM.pdf]

Supplements to

Estimating stimulus-processing time via position slopes in visual search

Supplement A: Instructions

Instructions of Experiment 1

The instructions for the simple- and complex-stimuli conditions were identical in wording, but differed in the illustrations of the corresponding stimuli. They were presented in white text on a gray background. Participants could advance to the next instruction page by pressing the spacebar.

General Instructions. The experiment began with a welcome message: “Herzlich Willkommen! In diesem Experiment wirst Du eine Suchaufgabe durchführen. Bitte lies Dir die folgende Anleitung aufmerksam durch.” (English: “Welcome! In this experiment, you will perform a search task. Please read the following instructions carefully.”). Each part of the experiment (simple- and complex-stimuli condition) started with an explanation of the search templates (Fig. A1A), the task (Fig. A1C), and the information that the target never appeared at the 12 o’clock position (Fig. A1B).

Figure A1

Instructions of Experiment 1 and 2

A

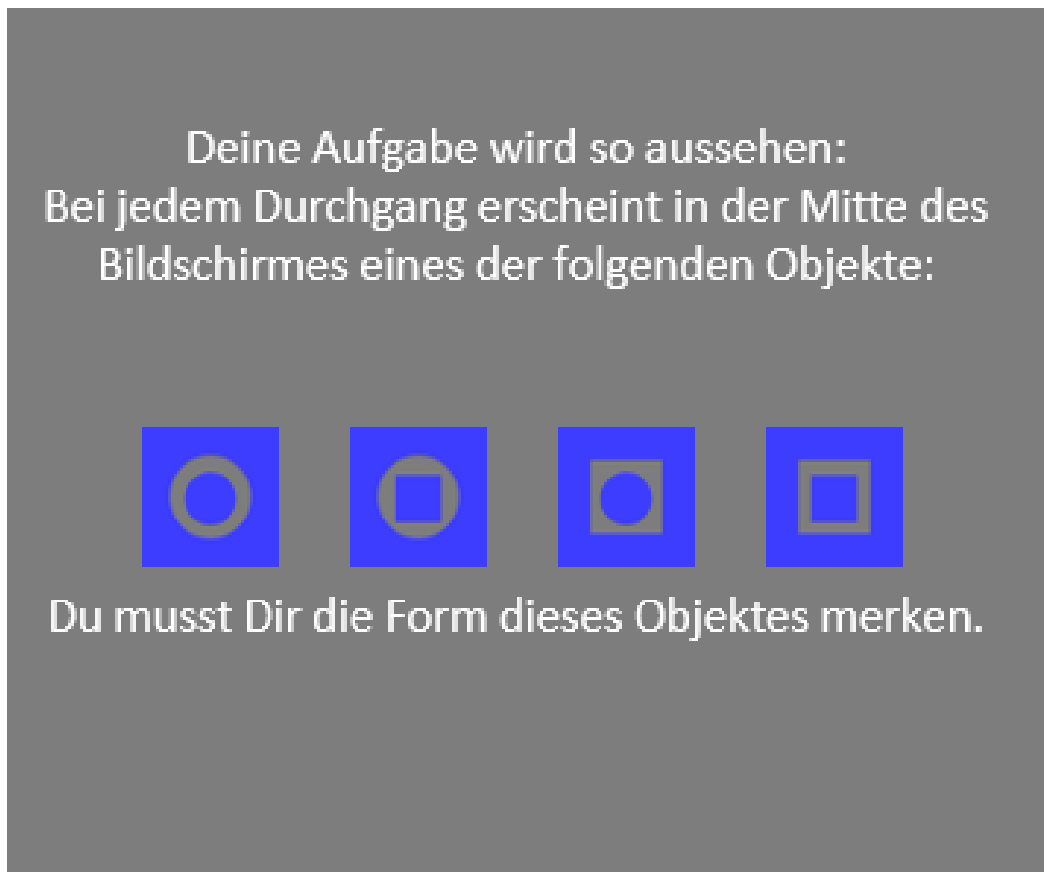

"Your task will be as follows: In each trial, one of the following objects will appear in the center of the screen: You must remember the shape of this object."

B

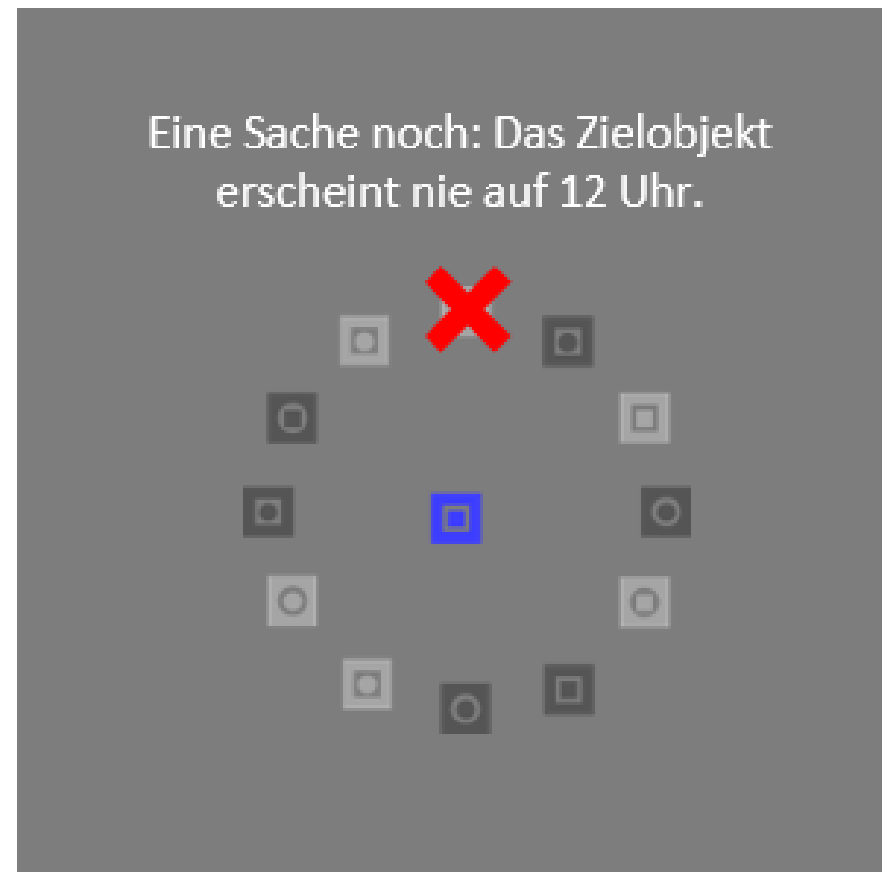

"One more thing: The target object never appears at the 12 o'clock position.“

C

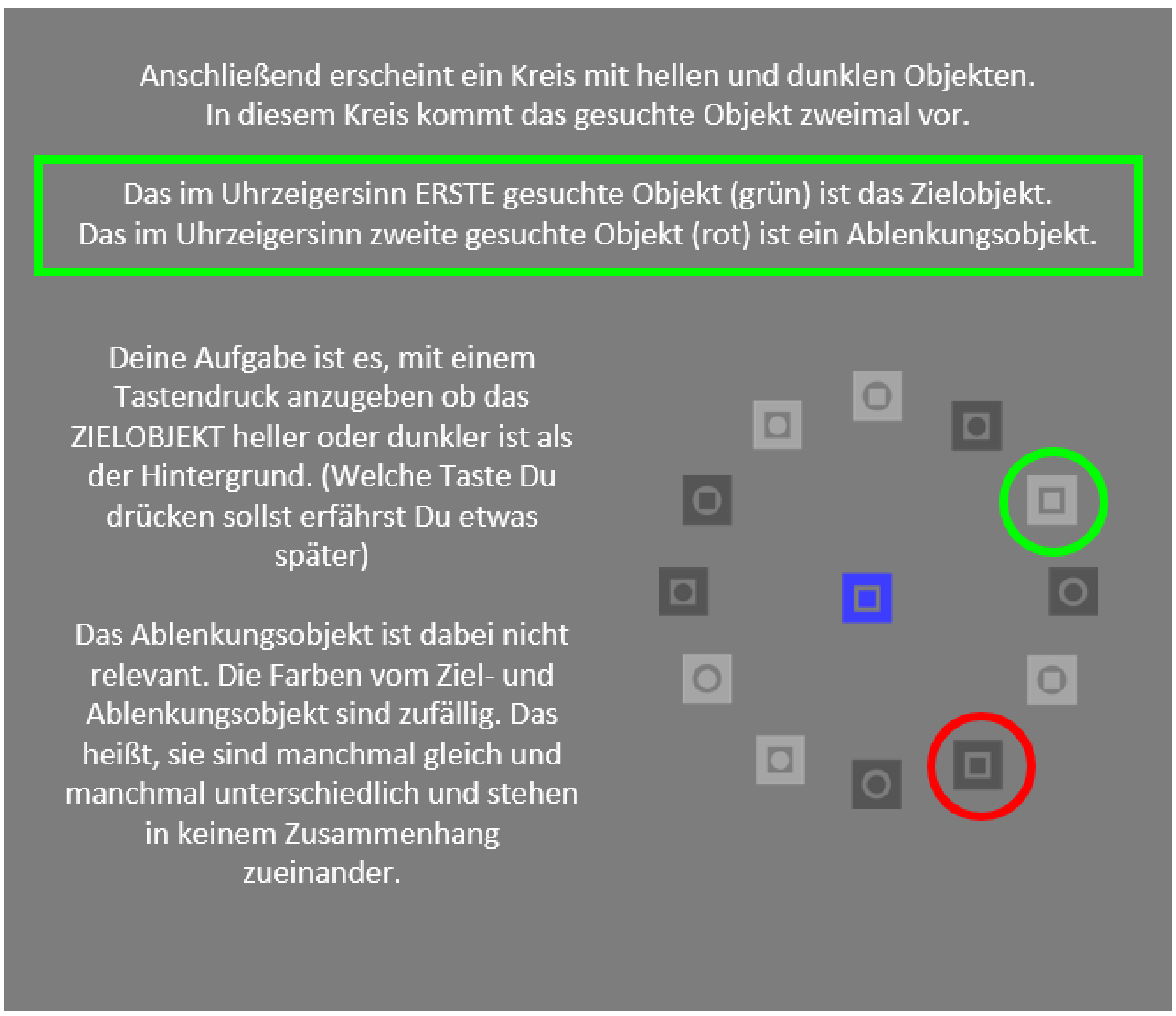

"Afterwards, a circle containing light and dark objects will appear. Within this circle, the searched object appears twice. The first searched object in clockwise order (green) is the target object. The second searched object in clockwise order (red) is a lure object. Your task is to indicate by pressing a key whether the TARGET OBJECT is lighter or darker than the background. (You will learn which key to press a little later.) The lure object is not relevant. The colors of the target and lure objects are random — this means they are sometimes the same and sometimes different, and they are not related to each other."

Note. The stimuli shown here are from the simple-stimuli condition of Experiment 1. Complex stimuli and the respective templates were shown in the complex condition of Experiment 1. Templates (Panel A) were identical in Experiment 2, but the search displays (Panels B and C) contained additional stimuli (see Fig. 2A).

Pretraining. The pretraining phase of each part was introduced with two text screens: "Um Dich mit der Aufgabe vertraut zu machen, wirst Du im Folgenden eine Trainingsaufgabe durchführen. Jetzt kannst Du prüfen, ob Du die Aufgabe richtig verstanden hast. Wenn Dir etwas unklar ist, melde Dich gerne bei der Versuchsleitung!" (English: "To help you become familiar with the task, you will now complete a short practice task. You can now check whether you have understood the task correctly. If anything is unclear, please feel free to ask the experimenter!") and "Jetzt möchten wir prüfen, ob klar geworden ist, welches das Zielobjekt ist. Bitte klicke im Folgenden mit der Maus immer nur auf das Zielobjekt." (English: "Now we would like to check whether it is clear which one is the target object. In the following, please click only on the target object using the mouse."). Participants then completed six pretraining trials. If more than one trial was answered incorrectly, the following message appeared: "Huch, das hat ja noch nicht so gut geklappt. Scheinbar konnten wir nicht deutlich beschreiben, was das Zielobjekt ist. Wende Dich bitte an die Versuchsleitung und lass Dir die Aufgabe nochmal erklären." (English: "Oops, that didn't work so well yet. Apparently, we didn't explain clearly enough what the target object is. Please contact the experimenter and have the task explained to you again."), and the pretraining was repeated. Otherwise, the message "Glückwunsch, Du weißt genau, was das Zielobjekt ist! Als nächstes kannst Du die eigentliche Aufgabe trainieren. Im kommenden Übungsblock sollst Du das Zielobjekt nicht mehr anklicken, sondern die Farbe des Zielobjektes angeben." (English: "Congratulations, you know exactly what the target object is! Next, you can practice the actual task. In the upcoming practice block, you no longer click on the target object but instead indicate the color of the target object.") was shown.

Training. The training phase began with information about the required response (Fig. A2). Participants were then instructed to position their fingers and start the training themselves: "So

weit alles klar? Dann lege jetzt Deine Zeigefinger auf die Tasten D und K. Starte den Übungsblock mit einer dieser Tasten." (English: "All clear so far? Then place your index fingers on the D and K keys. Start the practice block by pressing one of these keys.").

Figure A2
Response Instruction in Experiment 1

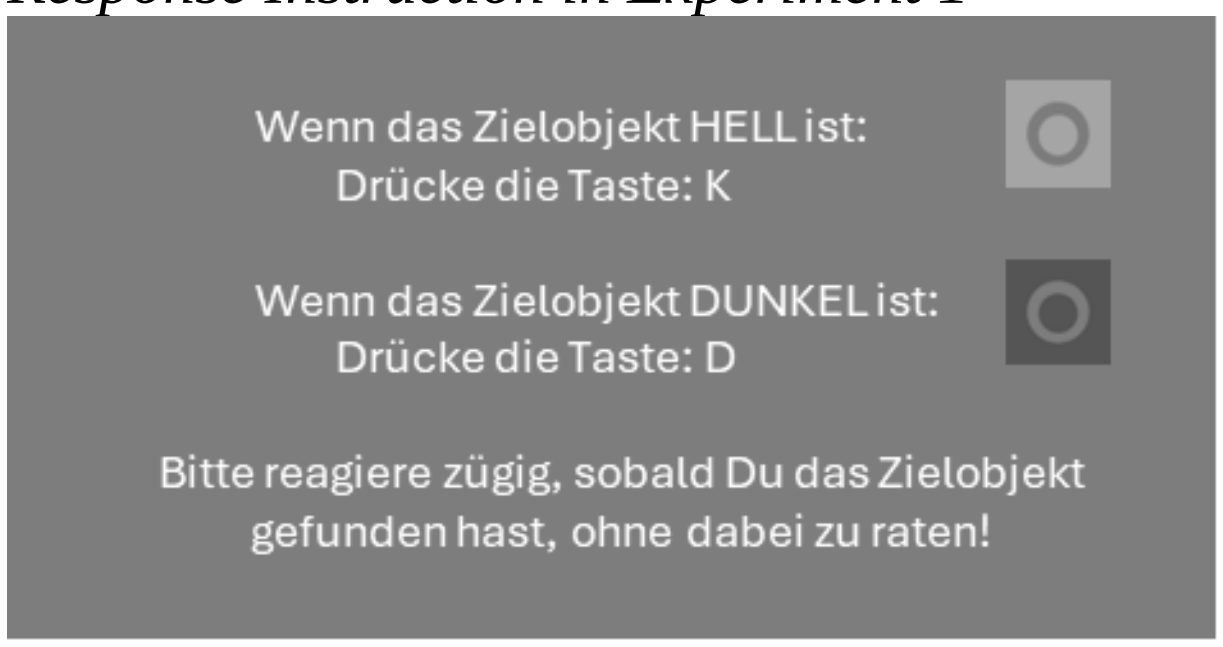

"If the target object is LIGHT: Press the K key. If the target object is DARK: Press the D key. Please respond quickly as soon as you have found the target object, but without guessing!"

Note. The figure shows one out of two possible response mappings and simple stimuli.

In the corresponding cases, an error message ("Leider falsch!"; English: "Unfortunately, wrong!") or a timeout message ("Leider zu langsam!"; English: "Unfortunately, too slow!") appeared in red text. At the end of the training, participants received feedback on their accuracy: "Das Training ist beendet. Deine Genauigkeit lag bei xx %." (English: "The training is complete. Your accuracy was xx %."). If accuracy was below 80 %, the following message appeared and the training was repeated: "Deine Genauigkeit lag unter 80 %. Scheinbar waren die Erklärungen nicht ganz deutlich. Bitte wende Dich an die Versuchsleitung, damit Du die Aufgabe noch einmal besprechen kannst. Anschließend wird der Trainingsblock wiederholt." (English: "Your accuracy was below 80 %. Apparently, the instructions were not entirely clear. Please contact the experimenter so that you can talk about the task again. Afterwards, the training block will be repeated.").

Experiment. The experimental blocks began with the message: "Sehr gut, Du hast auch das Training geschafft! Als nächstes startest Du mit dem eigentlichen Versuch. Du wirst nun in 3 Blöcken dieselbe Aufgabe wie im Training durchführen. Jeder Block dauert ungefähr 10-15

Minuten, aber Du hast alle paar Minuten die Möglichkeit eine Pause einzulegen, wenn Du möchtest.” (English: “Very good, you’ve completed the training! Next, you’ll begin the actual experiment. You will now perform 3 blocks of the same task as in the training. Each block takes about 10–15 minutes, and you’ll have the opportunity to take a short break every few minutes if you wish.”). Minor breaks were introduced with the message “Wenn Du willst, kannst du jetzt eine kleine Pause machen, um konzentriert zu bleiben! Lege anschließend Deine Zeigefinger auf die Tasten D und K. Drücke eine dieser Tasten, um weiterzumachen.” (English: “If you like, you can take a short break now to stay focused! Afterwards place your index fingers on the D and K keys. Press one of these keys to continue.”). Major breaks displayed the text “Das war der xx. Block! Deine Genauigkeit lag bei xx %.” (English: “That was block xx! Your accuracy was xx %.”). After each break, the following message was shown to prepare participants for the continuation of the task: “OK. Dann geht es jetzt weiter. Lege bitte Deine Zeigefinger auf die Tasten D und K. Drücke eine dieser Tasten, um weiterzumachen.” (English: “OK. Let’s continue. Please place your index fingers on the D and K keys. Press one of these keys to continue.”). The experiment ended with a farewell message in green text: “Geschafft! Danke, dass du bis zum Ende durchgehalten hast! Bitte wende Dich jetzt an die Versuchsleitung.” (English: “You did it! Thank you for sticking with it until the end! Please contact the experimenter now.”).

Instructions of Experiment 2

The instructions of Experiment 2 were largely identical to Experiment 1. The instructions for the simple and complex conditions were highly identical in wording and illustrations but differed in the description of the corresponding tasks.

General Instructions. The experiment began with the identical welcome message as in Experiment 1. Each part (simple- and complex-response condition) started with the same explanation of the search templates, the task, and the information that the target never appeared at the 12 o'clock position (see Fig. A1).

Pretraining. The pretraining phase was identical to Experiment 1, except for the stimuli.

Training. The training phase began with information about the required response (Figs. A3 and A4). In the simple-response condition the participants were then instructed: "Alles klar so weit? Dann kannst Du die Aufgabe als nächstes etwas üben. Lege jetzt Deine Finger auf die Tasten D und K. Starte den Übungsblock mit einer dieser Tasten." (English: "All clear so far? Then you can now practice the task a little. Place your fingers on the D and K keys. Start the practice block by pressing one of these keys."). In the complex condition the respective instruction was accompanied by an image as a reminder of the task (Fig. A4C).

Figure A3

Response Instructions in the Simple-response Condition of Experiment 2

A B

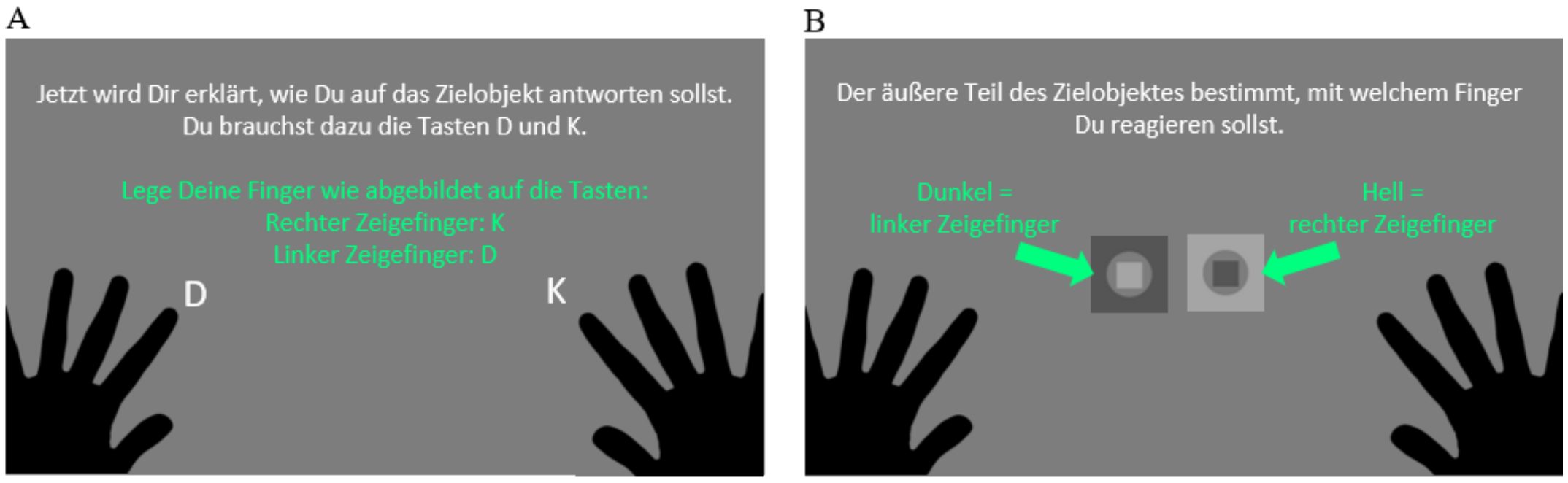

"You will now learn how to respond to the target object. For this, you will use the D and K keys. Place your fingers on the keys as shown: Right index finger: K; Left index finger: D."

"The outer part of the target object determines which finger you should respond with. Dark = left index finger; Light = right index finger."

Note. The figure shows one out of two possible mappings.

Experiment. The experimental blocks began with the same message as in Experiment 1. In the complex condition, before the start of each block a reminder of the required response was shown (Fig. A5). Minor breaks were introduced with the message "Wenn Du willst, kannst du

jetzt eine kleine Pause machen, um konzentriert zu bleiben! Drücke die LEERTASTE, wenn Du bereit bist weiterzumachen." (English: "If you like, you can take a short break now to stay focused! Press the SPACEBAR when you are ready to continue."). Major breaks were identical to Experiment1. The experiment ended with a farewell message identical to Experiment 1.

Figure A4

Response Instructions in the Complex-response Condition of Experiment 2

A

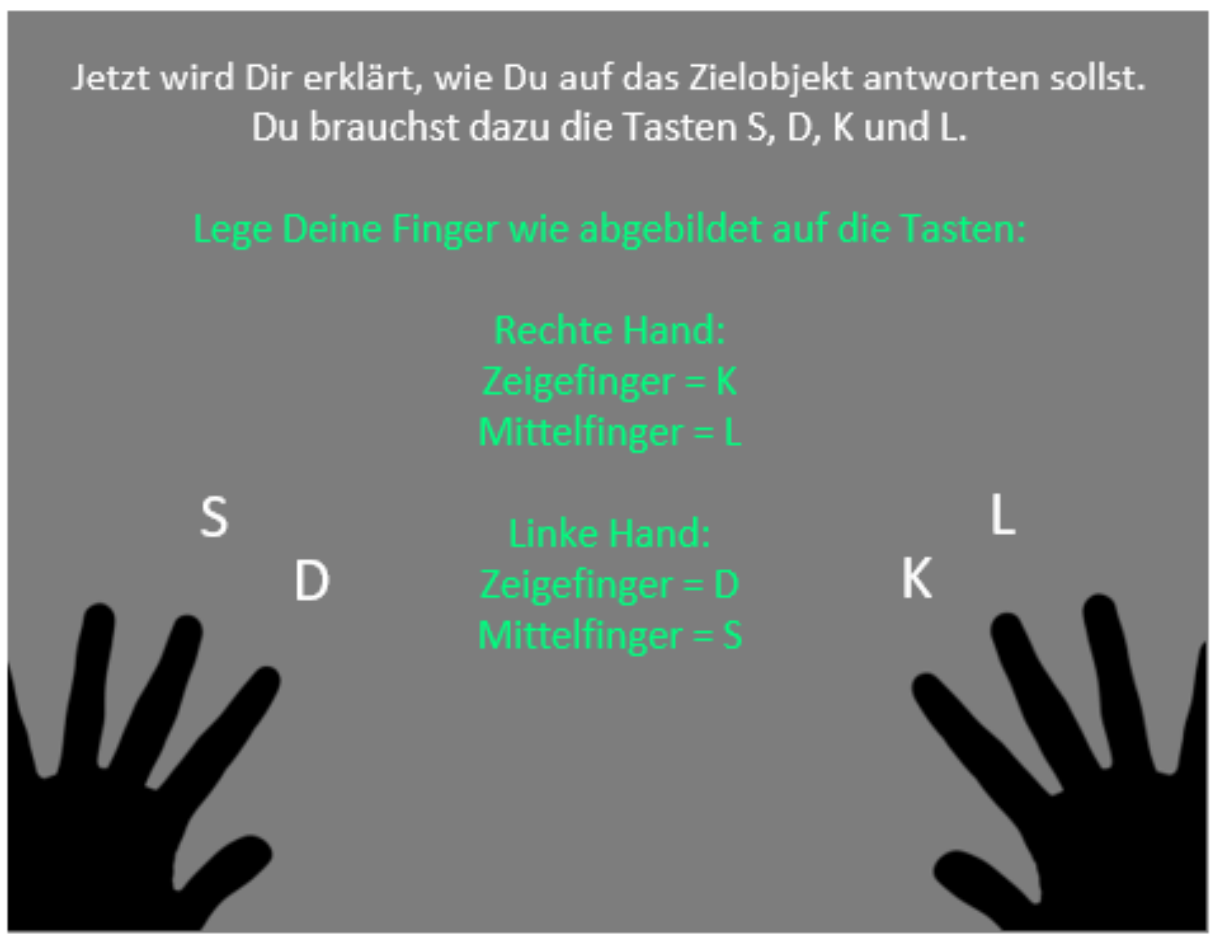

"You will now learn how to respond to the target object. For this, you will use the S, D, K and L keys. Place your fingers on the keys as shown: Right hand: Index finger = K; Middle finger = L; Left hand: Index finger = D; Middle finger = S."

B

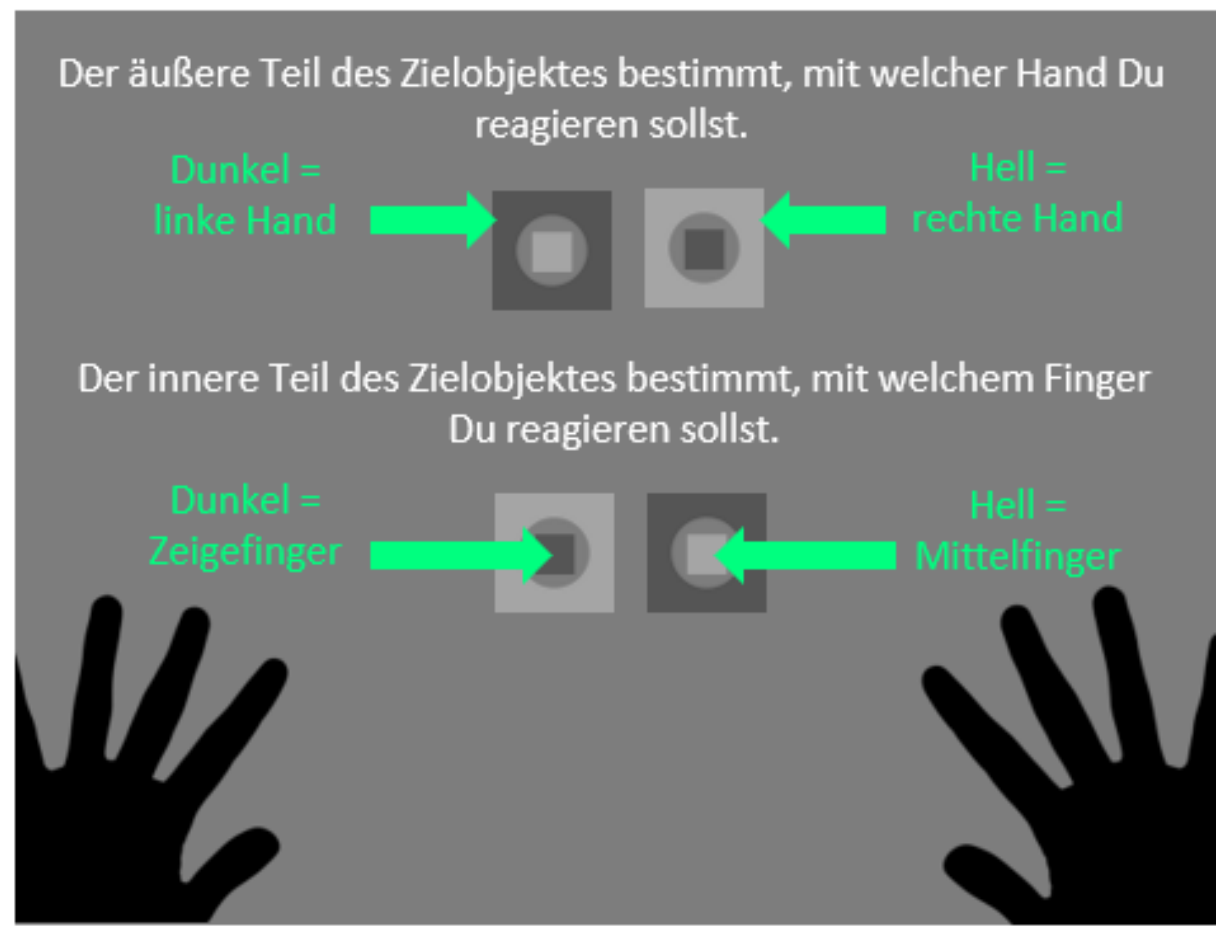

"The outer part of the target object determines which hand you should respond with. Dark = left hand; Light = right hand. The inner part of the target object determines which finger you should respond with. Dark = index finger; Light = middle finger."

C

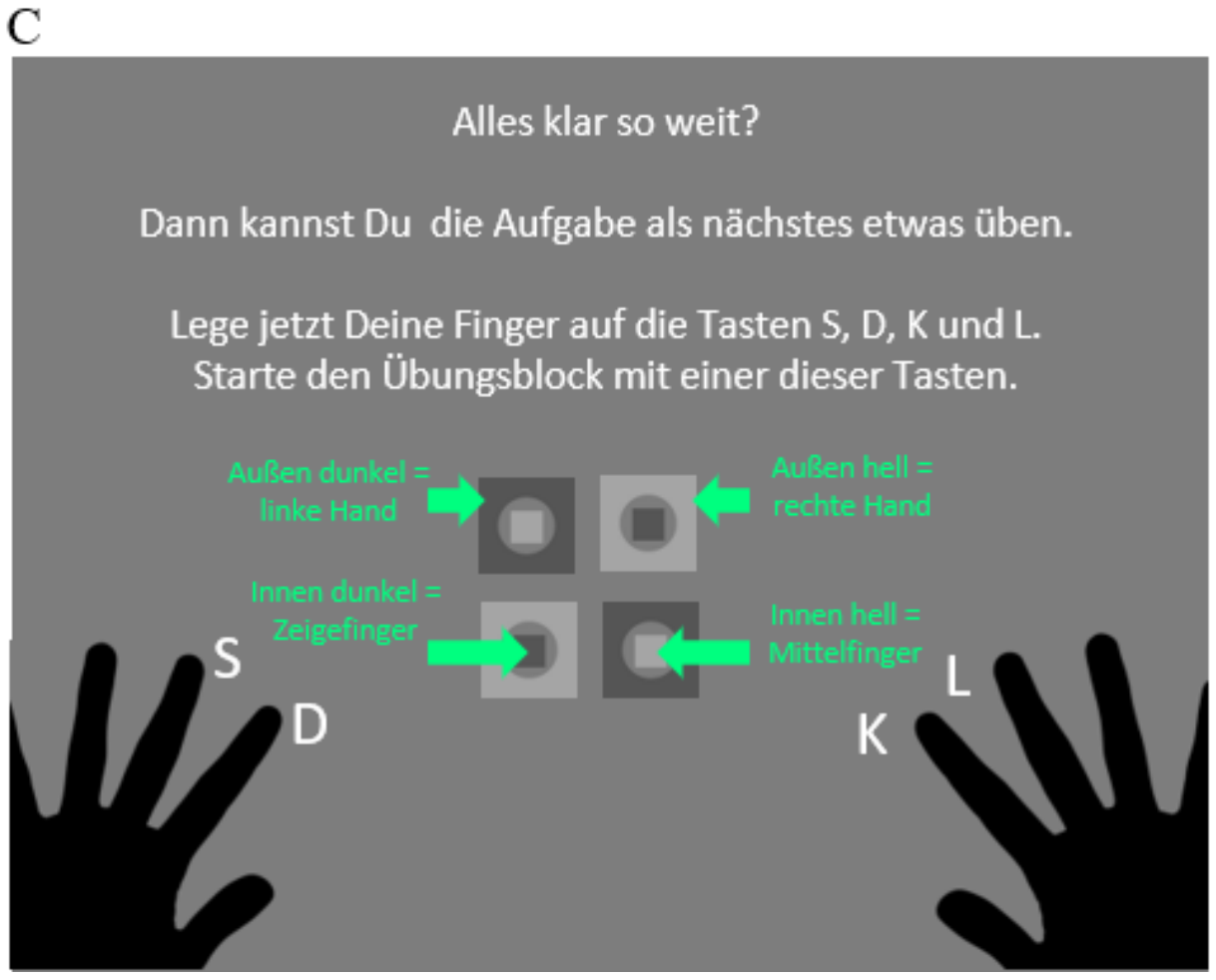

"All clear so far? Then you can now practice the task a little. Place your fingers on the S, D, K, and L keys. Start the practice block by pressing one of these keys. Outer part dark = left hand; Outer part light = right hand; Inner part dark = index finger; Inner part light = middle finger."

Note. The figure shows one out of four possible mappings.

Figure A5

Reminder of Response Instructions in the Complex-response Condition of Experiment 2

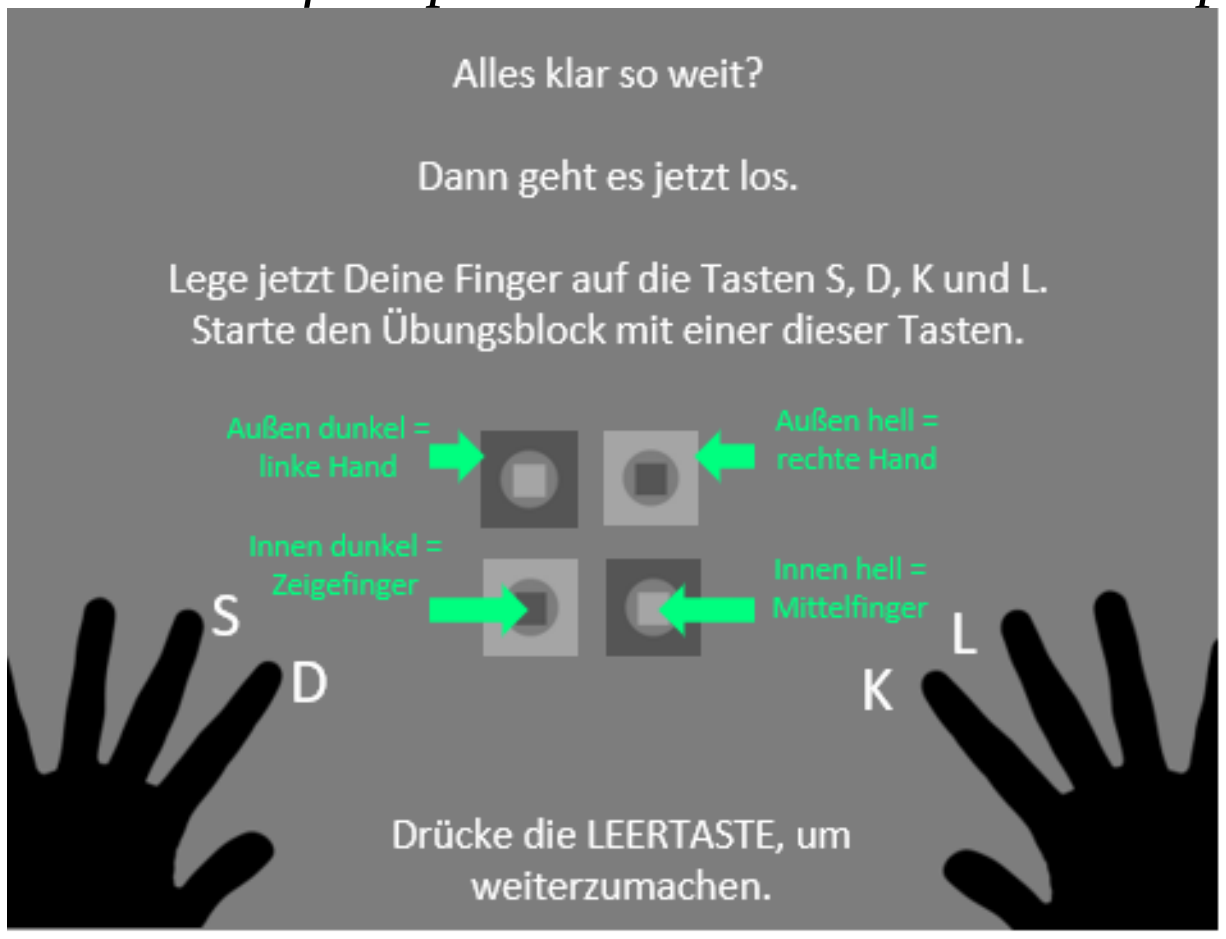

"All clear so far? Then let's get started. Place your fingers on the S, D, K, and L keys. Start the practice block by pressing one of these keys. Outer part dark = left hand; Outer part light = right hand; Inner part dark = index finger; Inner part light = middle finger."

Note. The reminder was only shown in the experimental blocks of the complex-response condition.

Supplement B: Individual position functions

Figure B1

Individual Position Functions in Experiment 1 Sorted by Block Order

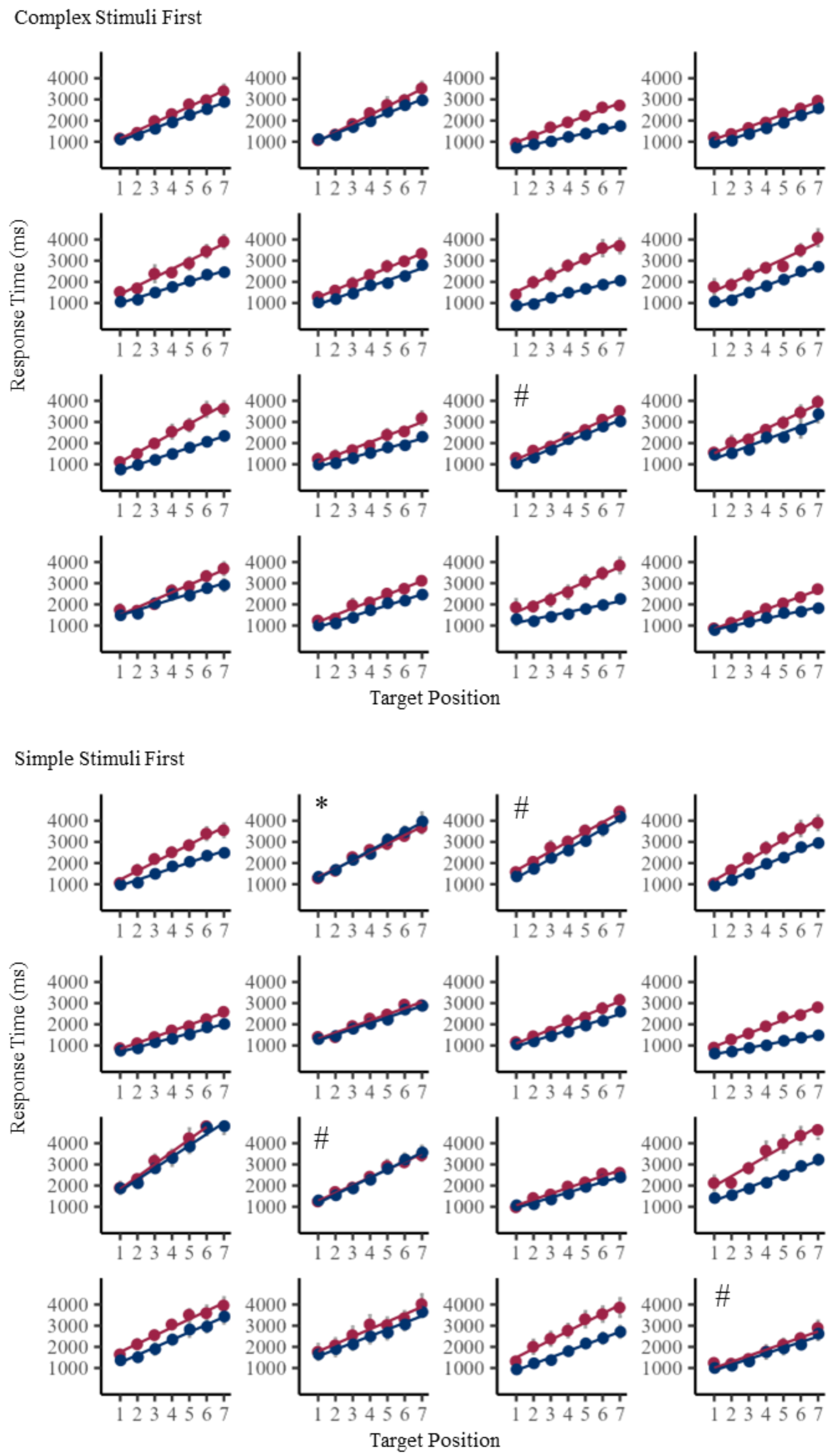

Note. Median RTs per stimulus condition and position for each participant (individual panels). Note that for almost all participants, complex stimuli (red lines) produce steeper slopes than simple stimuli (blue lines). Only one participant (marked with *) shows the reversed pattern and four participants (marked with #) show no effect.

Figure B2

Individual Position Functions in Experiment 2 Sorted by Block Order

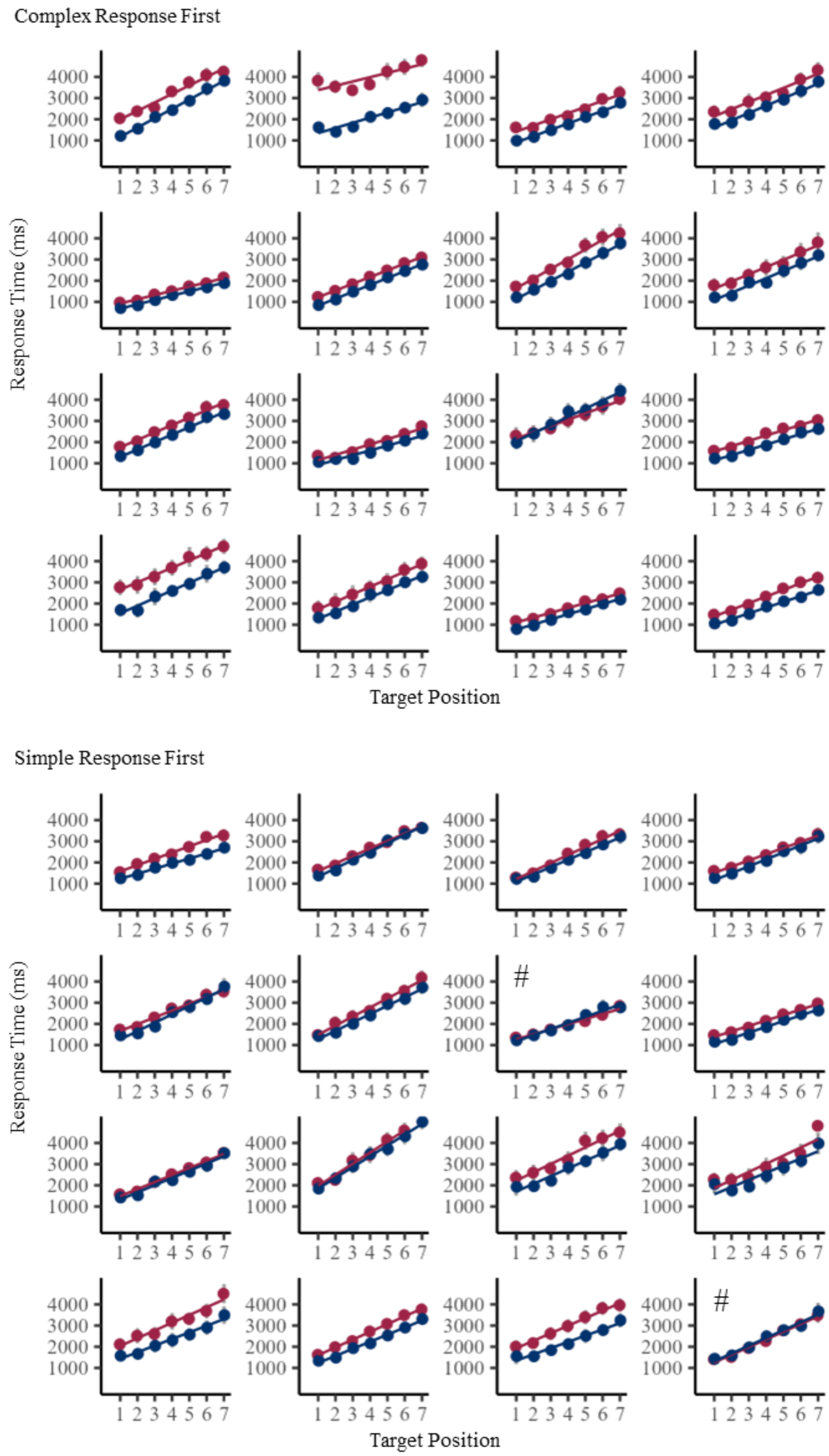

Note. Median RTs per response condition and position for each participant (individual panels). Note that the two lines are strikingly parallel and that for almost all participants, complex

responses (red lines) produce larger intercepts than simple responses (blue lines). Only two participants (marked with #) show no intercept effect.

Supplement C: Error rates

Following our hypotheses and pre-registrations, only RT results are reported in the main text. Here, we also report explorative analyses on error rates. Whereas overall, error rates were lower for simple (6.3 %; *SD* = 2.8) compared to complex stimuli (7.9 %; *SD* = 3.8) in Experiment 1, $t(31) = 3.01$, $p = .005$, $d = 0.53$, $BF_{10} = 1.02$, the intercepts (simple : 6.2 %, *SD* = 3.8 vs. complex: 6.6 %, *SD* = 3.8), $t(31) = 0.73$, $p = .470$, $d = 0.13$, $BF_{10} = .28$, and slopes (simple: 0.05%, *SD* = 0.92 vs. complex: 0.42 %, *SD* = 0.99), $t(31) = 1.52$, $p = .138$, $d = 0.27$, $BF_{10} = 0.68$, did not differ significantly (Fig. C1).

In Experiment 2, error rates were not significantly lower for simple (9.0 %; *SD* = 4.7) than complex responses (9.3 %; *SD* = 4.2), $t(31) = 0.51$, $p = .307$, $d = 0.09$, $BF_{10} = 0.26$, and the intercepts (simple: 10.8 %, *SD* = 7.0 vs. complex: 9.4 %, *SD* = 6.0), $t(31) = 1.50$, $p = .144$, $d = 0.26$, $BF_{10} = .35$, did not differ significantly either. While the slopes were steeper in the simple-response (-0.60 %, *SD* = 1.27) than in the complex-response condition (-0.03 %, *SD* = 1.23), $t(31) = 2.45$, $p = .020$, $d = 0.43$, $BF_{10} = 1.01$, both were negative (i.e., more errors with targets on earlier positions; Fig. C1). The negative slopes are mainly driven by the relatively high error rate for targets on Position 1, which might indicate that stimuli at that position are more likely to be skipped by some participants.

Figure C1
Error Rates of Experiment 1 and 2

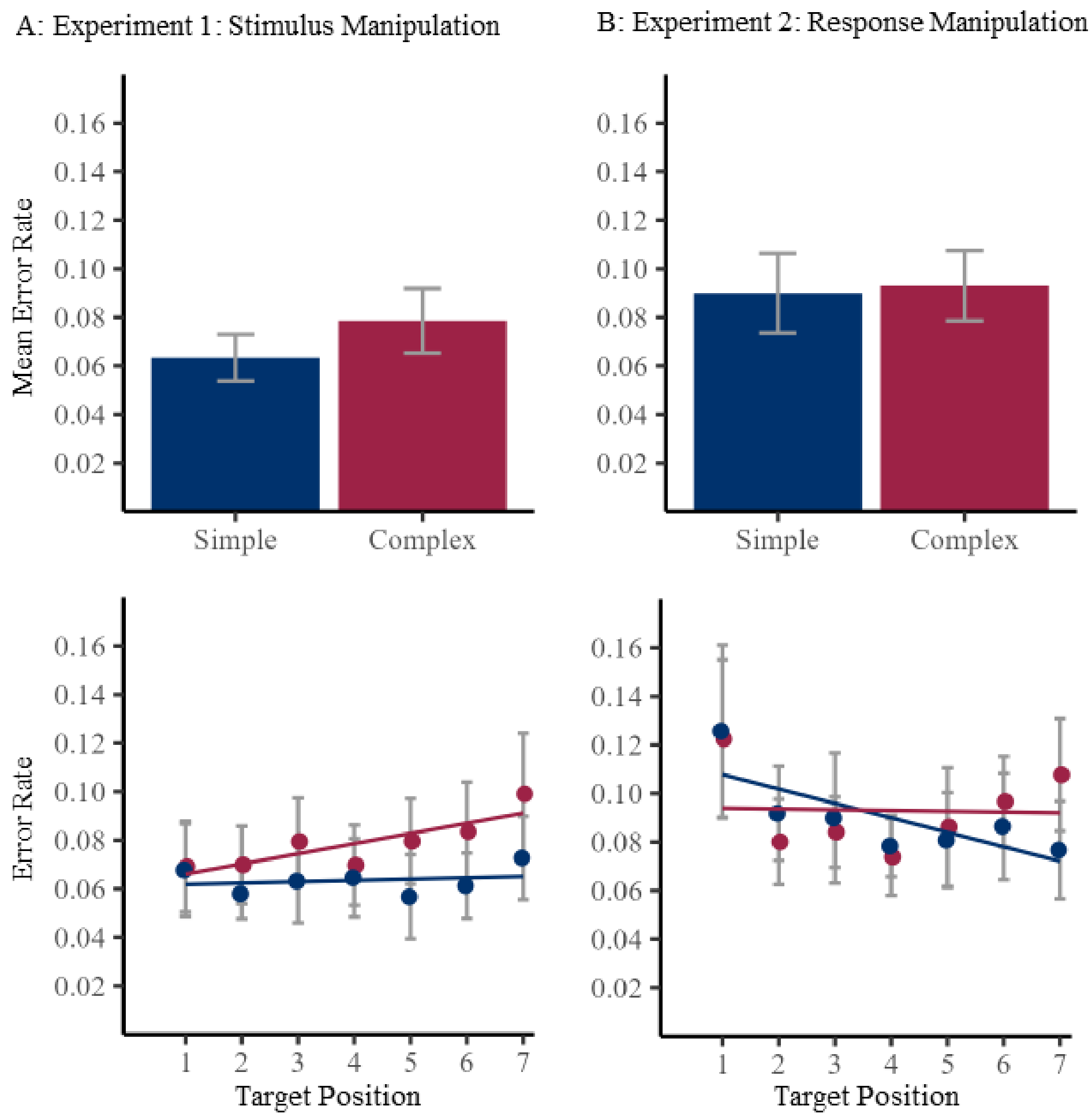

Note. Error bars indicate between-participants 95%-*CI*s.
